# Synthesis and preliminary structure-activity relationship study of 3-methylquinazolinone derivatives as EGFR inhibitors with enhanced antiproliferative activities against tumour cells

**DOI:** 10.1080/14756366.2021.1933466

**Published:** 2021-06-02

**Authors:** Yan Zhang, Qin Wang, Luolan Li, Yi Le, Li Liu, Jing Yang, Yongliang Li, Guochen Bao, Longjia Yan

**Affiliations:** a School of Pharmaceutical Sciences, Guizhou University, Guiyang, China; b State Key Laboratory of Functions and Applications of Medicinal Plants, Guizhou Medical University, Guiyang, China; c Guizhou Engineering Laboratory for Synthetic Drugs, Guiyang, China; d Shizhen College of Guizhou University of Traditional Chinese Medicine, Guiyang, China; e Faculty of Light Industry and Chemical Engineering, Guangdong University of Technology, Guangzhou, China; f Institute for Biomedical Materials and Devices (IBMD), Faculty of Science, University of Technology Sydney, Sydney, Australia

**Keywords:** Quinazolinone, EGFR, kinase inhibitor, structure-activity relationship, antiproliferative

## Abstract

In this paper, a set of 3-methylquniazolinone derivatives were designed, synthesised, and studied the preliminary structure-activity relationship for antiproliferative activities. All target compounds performed significantly inhibitory effects against wild type epidermal growth factor receptor tyrosine kinase (EGFR^wt^-TK) and tumour cells (A431, A549, MCF-7, and NCI-H1975). In particular, compound **4d** 3-fluoro-*N*-(4-((3-methyl-4-oxo-3,4-dihydroquinazolin-2-yl)methoxy)phenyl)benzamide showed higher antiproliferative activities against all tumour cells than Gefitinib (IC_50_ of 3.48, 2.55, 0.87 and 6.42 μM, respectively). Furthermore, compound **4d** could induce apoptosis of MCF-7 cells and arrest in G2/M phase at the tested concentration. Molecular docking and ADMET studies showed that compound **4d** could closely form many hydrogen bonds with EGFR^wt^-TK. Therefore, compound **4d** is potential to develop as novel anti-cancer drug.

## 1. Introduction

Epidermal growth factor receptor (EGFR) tyrosine kinase (TK), a receptor tyrosine kinase, plays an important role in the process of survival, proliferation, angiogenesis, tumour micro environment, and adhesion of several malignant tumors^1^. Overexpression or mutation of EGFR has been observed in many human tumour cells, such as nonsmall-cell lung cancer (NSCLC), breast cancer, and brain cancer^2^. Due to its crucial status related to cancer development, EGFR has been considered a potential drug target for many years^3–5^. Currently, a series of EGFR inhibitors were developed and approved into clinical phases, such as Gefitinib, Erlotinib, Icotinib, Afatinib, and Osimertinib^6^. However, with the continuous emergence of drug resistance in EGFR inhibitors in clinic, design and synthesis of novel EGFR inhibitors have become an urgent work for anti-tumour drug discovery^7^.

**Figure 1.**
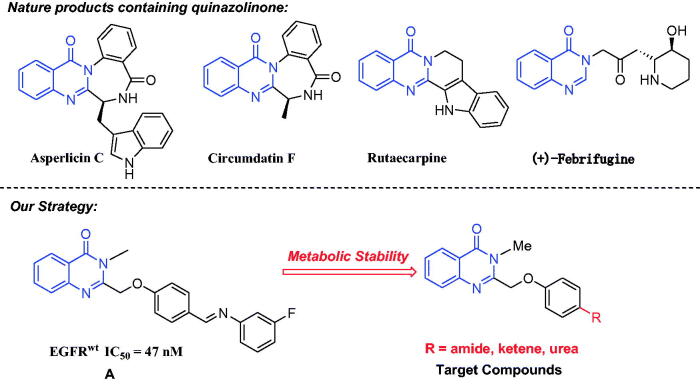
Natural products of quinazolinone and our strategy.

Quinazolone is the main skeleton of many natural products^8^^,^^9^, for example Allicin C, Cyclosporin F, Rutaecarpine, and (+)-Changshanine (Figure 1). These compounds have a wide range of biological activities, including antitumor, antibacterial, antiviral, and anti-inflammatory^10–12^. Therefore, many groups have developed new methods to synthesise quinazolone derivatives^13^. Patel et al. reported 2-phenylquinazolone derivatives as EGFR inhibitors in 2017^14^ and recently inspired from natural alkaloid L-norephedrine, Ghorab et al. published a 3-substituted quniazolinones for EGFR inhibitors^15^. These results indicate that the development of novel quinazolone EGFR inhibitors has attracted extensive attentions.

Our group has been focussed on the research of developing new anti-tumour drugs for several years^16–19^. We have synthesised lots of antiproliferative compounds such as benzodiazepines, 1,3,4-oxadiazoles, 1,3,4-thiadiazoles^20^, and pyrimidines^21^. Based on the broad activities of quinazolone and imperative need for EGFR inhibitors, we reported a series of 3-methylquinazolone derivatives for EGFR inhibitors in last year^22^. As shown in Figure 1, the IC_50_ value of compound **A** against EGFR^wt^ reached to 47 nm. However, further studies showed that compound **A** was not stable in mice plasma (Figure S1). The result prompted us to seek more valuable 3-methylquniazolinone derivatives. To overcome the metabolic stability in our previous research, we herein describe the design, synthesis, and studies the preliminary structure-activity relationship (SAR) of new 3-methylquinazolinone derivatives as EGFR inhibitors.

## 2. Experimental section

All reagents were commercially available in Sigma-Aldrich with analytical purity. Melting points were tested in digital melting point analyser with micro-display window (uncorrected, Shanghai Microelectronics Technology Co. Ltd.). The ^1^H and ^13 ^C NMR spectra were recorded on Bruker (Avance) 400 MHz and JEOL (Japan) 500 MHz NMR instrument with chemical reported as δ in CDCl_3_ and DMSO-d_6_, tetramethylsilane (TMS) as the internal standard. The high-resolution mass spectrometer (HRMS) was tested in TSQ 8000 high-resolution mass spectrometer and AB SCIEX X500R QTOF.

### 2.1. Synthesis

**Scheme 1.**
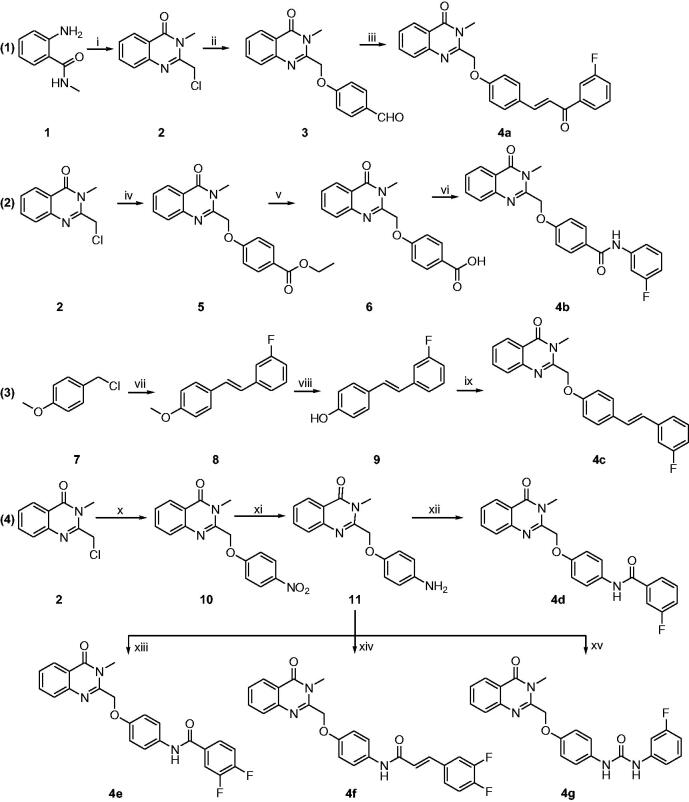
Synthetic route of compounds **4a-4g**. Reagents and conditions: i) 2-chloroacetyl chloride, AcOH, reflux, 50% yield; ii) 4-hydroxybenzaldehyde, K_2_CO_3_, KI, DMF, rt, 68% yield; iii) 3-fluorophenylethanone, NaOH, EtOH, rt, 42% yield; iv) ethyl 4-hydroxybenzoate, K_2_CO_3_, KI, DMF, rt, 64% yield; v) NaOH, EtOH, rt, 58% yield; vi) 3-fluorobenzenamine, HATU, DIEA, DMF, rt, 38% yield; vii) triethyl phosphate, tetrabutylammonium bromide, 130 °C, then 3-fluorobenzaldehyde, NaH, THF, rt, 43% yield; viii) BBr_3_, DCM, 0–10 °C, 72% yield; ix) compound **2**, K_2_CO_3_, KI, DMF, rt, 47% yield; x) 4-nitrophenol, K_2_CO_3_, KI, DMF, rt, 53% yield; xi) Pd/C, MeOH, H_2_, rt, 72% yield; xii–xv) corresponding acid or isocyanate, HATU, DIEA, DMF, rt, 30–37% yield.

The synthetic route of compounds **4a–4g** was shown in Scheme 1. The experimental methods and details were described as follows.

#### 2.1.1. 2-(Chloromethyl)-3-methylquinazolin-4(3H)-one (2)

2-Amino-*N*-methylbenzamide (1.5 g, 10 mmol) was dissolved in acetic acid (50 ml), and then 2-chloroacetyl chloride (3.36 g, 30 mmol) was added. The mixture was stirred for 6 h under refluxing. Then, the solution was evaporated *in vacuo* and neutralised with aqueous NaHCO_3_. The crude product was filtered and purified with recrystallisation from ethanol. The product was dried in vacuum to give a white solid. 1.04 g; 50% yield; ^1^H NMR (400 MHz, CDCl_3_) δ 8.29 (d, *J* = 8.0 Hz, 1H), 7.80 − 7.75 (m, 1H), 7.68 − 7.69 (m, 1H), 7.50 − 7.54 (m, 1H), 4.65 (s, 2H), 3.76 (s, 3H). Spectral properties were in accordance with the literature^23^.

#### 2.1.2. 4-((3-Methyl-4-oxo-3,4-dihydroquinazolin-2-yl)methoxy)benzaldehyde (3)

*p*-Hydroxybenzaldehyde (610 mg, 5 mmol) was dissolved in DMF (20 ml), and then anhydrous K_2_CO_3_ (759 mg, 5.5 mmol) was added. After stirred for 15 min at room temperature, the mixture was added compound **2** (1.04 g, 5 mmol) and KI (913 mg, 5.5 mmol). The solution was stirred for the other 2 h, and poured into water (40 ml). The crude product was filtered and purified with recrystallisation from ethanol. The product was dried in vacuum to give a light-yellow solid. 1 g; 68% yield; ^1^H NMR (DMSO, 400 MHz), δ 9.90 (s, 1H), 8.16 (dd, *J* = 8.0, 1.0 Hz, 1H), 7.90 (d, *J* = 8.8 Hz, 2H), 7.82 (ddd, *J* = 8.4, 7.2, 1.6 Hz, 1H), 7.63 (d, *J* = 7.6 Hz, 1H), 7.60–7.52 (m, 1H), 7.32 (d, *J* = 8.8 Hz, 2H), 5.45 (s, 2H), 3.62 (s, 3H). Spectral properties were in accordance with the literature^23^.

#### 2.1.3. (*E*)-2-((4–(3-(3-Fluorophenyl)-3-oxoprop-1-enyl)phenoxy)methyl)-3-methylquinazolin-4(3*H*)-one (4a)

1–(3-Fluorophenyl)ethanone (276 mg, 2 mmol) and 4-((3-methyl-4-oxo-3,4-dihydroquinazolin-2-yl)methoxy)benzaldehyde (588 mg, 2 mmol) were dissolved in EtOH (20 ml), and then 2 mol/l NaOH (1 ml) was added. The mixture was stirred for 6 h at room temperature. Then, the solution was neutralised with 2 mol/l HCl (1 ml). The crude product was filtered and purified by chromatography using PE/EtOAc = 1/1 as the fluent solvent. The product was dried in vacuum to give a white solid. 348 mg; 42% yield; m.p. 208–209 °C; ^1^H NMR (400 MHz, DMSO) δ 8.17 (d, *J* = 7.6 Hz, 1H), 8.02 − 7.75 (m, 7H), 7.67 − 7.52 (m, 4H), 7.22 (d, *J* = 8.4 Hz, 2H), 5.40 (s, 2H), 3.63 (s, 3H); ^13 ^C NMR (125 MHz, DMSO) δ 189.5, 161.8, 161.6, 160.5, 159.6, 152.7, 146.9, 145.1, 135.0, 134.5 (d, *J* = 8.5 Hz), 131.3, 130.9 (d, *J* = 3.0 Hz, 7H), 128.2, 127.9, 127.7, 126.8, 125.3 (d, *J* = 3.5 Hz), 124.2 (d, *J* = 3.5 Hz), 120.8, 117.1 (d, *J* = 22.0 Hz), 116.1, 69.0, 30.4; HRMS-ESI (*m/z*): [M + H]^+^ calcd for C_25_H_19_FN_2_O_3_, 415.1452; found, 415.1452.

#### 2.1.4. Ethyl 4-((3-methyl-4-oxo-3,4-dihydroquinazolin-2-yl)methoxy)benzoate (5)

Ethyl 4-hydroxybenzoate (830 mg, 5 mmol) was dissolved in DMF (20 ml), and then anhydrous K_2_CO_3_ (759 mg, 5.5 mmol) was added. After stirred for 15 min at room temperature, the mixture was added compound **2** (1.04 g, 5 mmol) and KI (913 mg, 5.5 mmol). The solution was stirred for the other 2 h, and poured into water (40 ml). The crude product was filtered and purified with recrystallisation from ethanol. The product was dried in vacuum to give a white solid. 1.08 g; 64% yiled; m.p. 114–116 °C; ^1^H NMR (400 MHz, CDCl_3_) δ 8.30 (d, *J* = 8.8 Hz, 1H), 8.03 (d, *J* = 8.8 Hz, 2H), 7.77 (t, *J* = 7.6 Hz, 1H), 7.71 (d, *J* = 7.6 Hz, 1H), 7.52 (t, *J* = 8.0 Hz, 1H), 7.10 (d, *J* = 8.8 Hz, 2H), 5.25 (s, 2H), 4.35 (q, *J* = 7.2 Hz, 2H), 3.74 (s, 3H), 1.38 (t, *J* = 7.2 Hz, 3H); ^13 ^C NMR (100 MHz, DMSO) δ 165.7, 162.0, 161.7, 152.5, 146.9, 134.9, 131.6, 127.8, 127.6 126.7, 123.4, 120.7, 115.5, 68.8, 60.9, 30.3, 14.7; HRMS-ESI (*m/z*): [M + H]^+^ calcd for C_19_H_18_N_2_O_4_, 339.1339; found, 339.1339.

#### 2.1.5. 4-((3-Methyl-4-oxo-3,4-dihydroquinazolin-2-yl)methoxy)benzoic acid (6)

Compound **5** (676 mg, 2 mmol) was dissolved in EtOH (10 ml), and then 2 mol/l NaOH (2 ml) was added. The mixture was stirred for 6 h at room temperature. Then, the solution was neutralised with 2 mol/l HCl (2 ml). The crude product was filtered and dried in vacuum to give a white solid. 360 mg; 58% yield; m.p. 97–99 °C; ^1^H NMR (400 MHz, DMSO) δ 12.69 (s, 1H), 8.16 (d, *J* = 7.6 Hz, 1H), 7.91 (d, *J* = 8.8 Hz, 2H), 7.83 (t, *J* = 8.0 Hz, 1H), 7.65 (d, *J* = 8.4 Hz, 1H), 7.57 (t, *J* = 7.6 Hz, 1H), 7.20 (d, *J* = 8.8 Hz, 2H), 5.39 (s, 2H), 3.61 (s, 3H); ^13 ^C NMR (100 MHz, DMSO) δ 167.4, 161.7, 152.6, 146.9, 134.9, 131.8, 127.8, 127.6, 126.7, 124.3, 120.7, 115.3, 68.8, 30.3; HRMS-ESI (*m/z*): [M + H]^+^ calcd for C_17_H_14_N_2_O_4_, 311.1036; found, 311.1023.

#### 2.1.6. *N*-(3-Fluorophenyl)-4-((3-methyl-4-oxo-3,4-dihydroquinazolin-2-yl)methoxy)benzamide (4 b)

Compound **6** (310 mg, 1 mmol) and 3-fluorobenzenamine (111 mg, 1 mmol) were dissolved in DMF (5 ml), and then HATU (o-(7-aza-1H-benzotriazol-1-yl)-*N,N,N',N'*-tetramethyluronium hexafluorophosphate) (380 mg, 1 mmol) was added. After stirred for 30 min, the mixture was added DIEA (*N,N*-diisopropylethylamine) (129 mg, 1 mmol) and stirred for 12 h at room temperature. The solution was poured into water (20 ml). The final product was filtered and dried in vacuum to give a white solid. 153 mg; 38% yield; m.p. 196–197 °C; ^1^H NMR (400 MHz, DMSO) δ 10.28 (s, 1H), 8.16 (d, *J* = 8.0 Hz, 1H), 7.96 (d, *J* = 8.8 Hz, 2H), 7.83 (t, *J* = 7.6 Hz, 1H), 7.74 (d, *J* = 12.0 Hz, 1H), 7.65 (d, *J* = 8.0 Hz, 1H), 7.56 (dd, *J* = 14.0, 7.6 Hz, 2H), 7.37 (dd, *J* = 15.6, 8.0 Hz, 1H), 7.26 (d, *J* = 8.8 Hz, 2H), 6.91 (td, *J* = 8.4, 2.4 Hz, 1H), 5.42 (s, 2H), 3.63 (s, 3H); ^13 ^C NMR (125 MHz, DMSO) δ 152.5, 150.8, 149.4 (d, *J* = 19.0 Hz), 148.8, 142.2, 137.5, 133.3 (d, *J* = 9.0 Hz), 128.0, 124.6 (d, *J* = 7.5 Hz), 124.1, 122.4 (d, *J* = 15.5 Hz), 122.1, 121.4, 116.6, 113.1, 112.2, 108.3 (d, *J* = 16.5 Hz), 105.9, 105.8, 75.1, 44.3; HRMS-ESI (*m/z*): [M + H]^+^ calcd for C_23_H_18_FN_3_O_3_, 404.1404; found, 404.1405.

#### 2.1.7. (*E*)-1-(4-Methoxystyryl)-3-fluorobenzene (8)

1-(Chloromethyl)-4-methoxybenzene (1.56 g, 10 mmol) was dissolved in triethyl phosphate (50 ml), and then tetrabutylammonium bromide (322 mg, 1 mmol) was added. The mixture was stirred at 130 °C under argon for overnight. After evaporated under vacuum, the solution was added to the solution of 3-fluorobenzaldehyde (1.24 g, 10 mmol) in anhydrous THF (50 ml). The mixture was added NaH (600 mg, 15 mmol, 60% in oil) at 0–10 °C and stirred for 12 h at room temperature. The solution was added to water (100 ml) and extracted with EtOAc (100 ml × 2), and dried over MgSO_4_ for 6 h. The crude product was evaporated and purified with flash chromatography using PE/EtOAc = 1/1 as the fluent solvent. The product was dried in vacuum to give a light solid. 980 mg; 43% yield; ^1^H NMR (400 MHz, CDCl_3_) δ 7.47 − 7.42 (m, 2H), 7.28 (dd, *J* = 8.0, 6.0 Hz, 1H), 7.24 − 7.16 (m, 2H), 7.05 (d, *J* = 16.4 Hz, 1H), 6.94 − 6.87 (m, 4H), 3.83 (s, 3H). Spectral properties were in accordance with the literature^24^.

#### 2.1.8. (*E*)-4-(3-Fluorostyryl)phenol (9)

Compound **8** (1.14 g, 5 mmol) was dissolved in DCM (10 ml), and then 1 mol/l BBr_3_ (10 ml) in THF was added at 0–10 °C. The mixture was stirred at room temperature for 6 h. The solution was evaporated under vacuum. The crude product was filtered and purified with recrystallisation from ethanol. The product was dried in vacuum to give a white solid. 770 mg; 72% yield; ^1^H NMR (400 MHz, CDCl_3_) δ 7.43 − 7.39 (m, 2H), 7.30 (td, *J* = 8.0, 6.0 Hz, 1H), 7.24 − 7.16 (m, 2H), 7.05 (d, *J* = 16.4 Hz, 1H), 6.95 − 6.89 (m, 2H), 6.86 − 6.82 (m, 2H), 4.83 (s, 1H). Spectral properties were in accordance with the literature^25^.

#### 2.1.9. (*E*)-2-((4-(3-Fluorostyryl)phenoxy)methyl)-3-methylquinazolin-4(3*H*)-one (4c)

Compound **9** (214 mg, 1 mmol) was dissolved in DMF (5 ml), and then anhydrous K_2_CO_3_ (152 mg, 1.1 mmol) was added. After stirred for 15 min at room temperature, the mixture was added compound **2** (208 g, 1 mmol) and KI (183 mg, 1.1 mmol). The solution was stirred for the other 2 h, and poured into water (40 ml). The crude product was filtered and purified with recrystallisation from ethanol. The product was dried in vacuum to give a white solid. 181 mg; 47% yield; m.p. 160–162 °C; ^1^H NMR (400 MHz, CDCl_3_) δ 8.30 (d, *J* = 8.0 Hz, 1H), 7.79 − 7.70 (m, 2H), 7.54 − 7.45 (m, 3H), 7.29 (dd, *J* = 8.0, 6.0 Hz, 1H), 7.20 (dd, *J* = 20.0, 9.2 Hz, 2H), 7.05 (dd, *J* = 12.8, 6.4 Hz, 3H), 6.93 (dd, *J* = 9.2, 7.2 Hz, 2H), 5.21 (s, 2H), 3.75 (s, 3H); ^13 ^C NMR (125 MHz, DMSO) δ 151.3, 149.7, 149.5, 146.5, 142.4, 137.6, 132.4 (d, *J* = 6.5 Hz), 128.0, 124.8 (d, *J* = 6.5 Hz), 124.6, 124.0, 122.8, 122.2 (d, *J* = 19.5 Hz), 121.4, 120.7, 118.5, 116.6, 112.7, 111.51 (d, *J* = 17.0 Hz), 110.2 (d, *J* = 17.0 Hz), 75.4, 44.4; HRMS-ESI (*m/z*): [M + H]^+^ calcd for C_24_H_19_FN_2_O_2_, 387.1503; found, 387.1499.

#### 2.1.10. 3-Methyl-2-((4-nitrophenoxy)methyl)quinazolin-4(3*H*)-one (10)

4-Nitrophenol (695 mg, 5 mmol) was dissolved in DMF (20 ml), and then anhydrous K_2_CO_3_ (759 mg, 5.5 mmol) was added. After stirred for 15 min at room temperature, the mixture was added compound **2** (1.04 g, 5 mmol) and KI (915 mg, 5.5 mmol). The solution was stirred for the other 2 h, and poured into water (80 ml). The crude product was filtered and purified with recrystallisation from ethanol. The product was dried in vacuum to give a yellow solid. 824 mg; 53% yield; m.p. 185–186 °C; ^1^H NMR (400 MHz, CDCl_3_) δ 8.32 (dd, *J* = 8.0, 1.2 Hz, 1H), 8.27 − 8.23 (m, 2H), 7.81 (ddd, *J* = 8.4, 7.2, 1.6 Hz, 1H), 7.74 (dd, *J* = 8.0, 0.8 Hz, 1H), 7.56 (ddd, *J* = 8.0, 7.2, 1.2 Hz, 1H), 7.23 − 7.19 (m, 2H), 5.33 (s, 2H), 3.77 (s, 3H); ^13 ^C NMR (125 MHz, DMSO) δ 150.9, 149.4, 141.8, 137.5, 133.5, 128.0, 122.3, 122.1, 121.4, 121.0, 116.6, 112.9, 75.0, 44.1; HRMS-ESI (*m/z*): [M + H]^+^ calcd for C_16_H_13_N_3_O_4_, 312.0978; found, 312.0982.

#### 2.1.11. 2-((4-Aminophenoxy)methyl)-3-methylquinazolin-4(3H)-one (11)

Compound **10** (622 mg, 2 mmol) was dissolved in MeOH (10 ml), and then Pd/C (62 mg, 10%) was added. The mixture was stirred at room temperature under hydrogen atmosphere for overnight. The solution was filtered with celite and the filtration was evaporated under vacuum. The crude product was purified with recrystallisation from MeOH. The product was dried in vacuum to give a light-yellow solid. 405 mg; 72% yield; m.p. 165–166 °C; ^1^H NMR (400 MHz, CDCl_3_) δ 8.29 (d, *J* = 8.8 Hz, 1H), 7.78 − 7.68 (m, 2H), 7.53 − 7.48 (m, 1H), 6.90 − 6.85 (m, 2H), 6.67 − 6.62 (m, 2H), 5.09 (s, 2H), 3.75 (s, 3H), 3.49 (s, 2H); ^13 ^C NMR (125 MHz, DMSO) δ 149.5, 142.8, 139.4, 137.6, 135.2, 128.0, 122.3, 122.1, 121.4, 116.6, 113.4, 112.2, 76.6, 44.6; HRMS-ESI (*m/z*): [M + H]^+^ calcd for C_16_H_15_N_3_O_2_, 282.1236; found, 282.1241.

#### 2.1.12. 3-Fluoro-N-(4-((3-methyl-4-oxo-3,4-dihydroquinazolin-2-yl)methoxy)phenyl)benzamide (4d)

Compound **11** (281 mg, 1 mmol) and 3-fluorobenzoic acid (140 mg, 1 mmol) were dissolved in DMF (5 ml), and then HATU (o-(7-aza-1H-benzotriazol-1-yl)-*N,N,N',N'*-tetramethyluronium hexafluorophosphate) (380 mg, 1 mmol) was added. After stirred for 30 min, the mixture was added DIEA (*N,N*-diisopropylethylamine) (129 mg, 1 mmol) and stirred for 12 h at room temperature. The solution was poured into water (20 ml). The final product was filtered and dried in vacuum to give a white solid. 149 mg; 37% yield; m.p. 228–229 °C; ^1^H NMR (400 MHz, DMSO) δ 10.23 (s, 1H), 8.17 (dd, *J* = 8.0, 1.2 Hz, 1H), 7.86 − 7.79 (m, 2H), 7.77 − 7.73 (m, 1H), 7.71 − 7.67 (m, 3H), 7.57 (dt, *J* = 2.0, 1.6 Hz, 2H), 7.44 (td, *J* = 8.4, 2.4 Hz, 1H), 7.15 − 7.10 (m, 2H), 5.29 (s, 2H), 3.64 (s, 3H); ^13 ^C NMR (125 MHz, DMSO) δ 151.4, 150.7, 149.5, 149.2, 143.6, 142.5, 137.6, 130.2 (d, *J* = 5.5 Hz), 128.0, 126.7, 124.9 (d, *J* = 6.5 Hz), 122.2 (d, *J* = 17.0 Hz), 121.4, 119.5, 118.0, 116.6, 115.1 (d, *J* = 16.5 Hz), 112.5, 111.9 (d, *J* = 18.0 Hz), 75.6, 44.4; HRMS-ESI (*m/z*): [M + Na]^+^ calcd for C_23_H_17_FN_3_NaO_3_, 426.1224; found, 426.1226.

#### 2.1.13. 3,4-Difluoro-N-(4-((3-methyl-4-oxo-3,4-dihydroquinazolin-2-yl)methoxy)phenyl)benzamide (4e)

Compound **11** (281 mg, 1 mmol) and 3,4-difluorobenzoic acid (158 mg, 1 mmol) were dissolved in DMF (5 ml), and then HATU (o-(7-aza-1H-benzotriazol-1-yl)-*N,N,N',N'*-tetramethyluronium hexafluorophosphate) (380 mg, 1 mmol) was added. After stirred for 30 min, the mixture was added DIEA (*N,N*-diisopropylethylamine) (129 mg, 1 mmol) and stirred for 12 h at room temperature. The solution was poured into water (20 ml). The final product was filtered and dried in vacuum to give a white solid. 139 mg; 33% yield; m.p. 248–249 °C; ^1^H NMR (400 MHz, DMSO) δ 10.23 (s, 1H), 8.16 (dd, *J* = 8.0, 1.2 Hz, 1H), 8.05 − 7.98 (m, 1H), 7.84 (dd, *J* = 12.0, 4.8 Hz, 2H), 7.70 − 7.55 (m, 5H), 7.13 (d, *J* = 9.2 Hz, 2H), 5.28 (s, 2H), 3.63 (s, 3H); ^13 ^C NMR (125 MHz, DMSO) δ 152.1, 150.7, 149.5, 143.7, 142.5, 137.6, 128.0, 126.6, 126.2 (d, *J* = 6.0 Hz), 122.2 (d, *J* = 18.0 Hz), 121.4, 120.5 (d, *J* = 2.0 Hz), 118.0, 116.6, 114.6, 114.5, 114.1, 114.0, 112.5, 75.6, 44.4; HRMS-ESI (*m/z*): [M + Na]^+^ calcd for C_23_H_16_F_2_N_3_NaO_3_, 444.1130; found, 444.1126.

#### 2.1.14. (*E*)-3-(3,4-Difluorophenyl)-*N*-(4-((3-methyl-4-oxo-3,4-dihydroquinazolin-2-yl)methoxy)phenyl)acrylamide (4f)

Compound **11** (281 mg, 1 mmol) and (*E*)-3–(3,4-difluorophenyl)acrylic acid (184 mg, 1 mmol) were dissolved in DMF (5 ml), and then HATU (o-(7-aza-1H-benzotriazol-1-yl)-*N,N,N',N'*-tetramethyluronium hexafluorophosphate) (380 mg, 1 mmol) was added. After stirred for 30 min, the mixture was added DIEA (*N,N*-diisopropylethylamine) (129 mg, 1 mmol) and stirred for 12 h at room temperature. The solution was poured into water (20 ml). The final product was filtered and dried in vacuum to give a white solid. 134 mg; 30% yield; m.p. 235–236 °C; ^1^H NMR (400 MHz, DMSO) δ 10.16 (s, 1H), 8.16 (d, *J* = 8.0 Hz, 1H), 7.86 − 7.82 (m, 1H), 7.75 − 7.63 (m, 4H), 7.59 − 7.49 (m, 4H), 7.11 (d, *J* = 9.1 Hz, 2H), 6.78 (d, *J* = 15.7 Hz, 1H), 5.27 (s, 2H), 3.63 (s, 3H); ^13 ^C NMR (125 MHz, DMSO) δ 150.6, 149.5, 143.4, 142.5, 137.6, 130.5, 128.0, 127.0, 126.6 (d, *J* = 5.0 Hz), 122.3, 122.1, 121.4, 120.2 (d, *J* = 2.5 Hz), 119.4, 118.2, 116.9, 116.6, 114.9 (d, *J* = 14.0 Hz), 113.6 (d, *J* = 14.0 Hz), 113.3, 112.6, 75.7, 44.5; HRMS-ESI (*m/z*): [M + Na]^+^ calcd for C_25_H_18_F_2_N_3_NaO_3_, 470.1287; found, 470.1288.

#### 2.1.15. 1-(3-Fluorophenyl)-3-(4-((3-methyl-4-oxo-3,4-dihydroquinazolin-2-yl)methoxy)phenyl)urea (4 g)

Compound **11** (281 mg, 1 mmol) was dissolved in anhydrous THF (5 ml), and then 1-fluoro-3-isocyanatobenzene (137 mg, 1 mmol) was added. After stirred for 30 min, the mixture was added DIEA (129 mg, 1 mmol) and stirred for 12 h at room temperature. The solution was poured into water (20 ml). The crude product was purified with recrystallisation from MeOH. The product was dried in vacuum to give a white solid. 142 mg; 34% yield; m.p. 195–196 °C; ^1^H NMR (400 MHz, DMSO) δ 9.13 (s, 1H), 8.84 (s, 1H), 8.16 (dd, *J* = 8.0, 1.2 Hz, 1H), 7.87 − 7.80 (m, 1H), 7.68 (d, *J* = 8.0 Hz, 1H), 7.57 (t, *J* = 7.6 Hz, 1H), 7.48 (dt, *J* = 12.0, 2.4 Hz, 1H), 7.39 (d, *J* = 9.2 Hz, 2H), 7.28 (dd, *J* = 15.2, 8.0 Hz, 1H), 7.12 (dd, *J* = 8.4, 0.8 Hz, 1H), 7.06 (d, *J* = 9.2 Hz, 2H), 6.75 (td, *J* = 8.4, 2.4 Hz, 1H), 5.24 (s, 2H), 3.63 (s, 3H); ^13 ^C NMR (125 MHz, DMSO) δ 151.1, 149.5 (d, *J* = 10.0 Hz), 142.6, 142.5, 137.6, 134.0 (d, *J* = 9.5 Hz), 128.0, 127.3, 124.6 (d, *J* = 8.0 Hz), 122.3, 122.2, 121.4, 116.6, 116.4, 112.7, 111.4, 106.6 (d, *J* = 17.0 Hz), 104.2, 104.0, 75.8, 44.5; HRMS-ESI (*m/z*): [M + Na]^+^ calcd for C_23_H_18_FN_4_NaO_3_, 441.1333; found, 441.1333.

### 2.2. *In vitro* EGFR^wt^-TK assay

Enzymatic inhibition of synthesised compounds against wild type EGFR tyrosine kinase was determined with enzyme-linked immunosorbent assays (ELISAs) as our previous methods. The recombinant EGFR^wt^-TK and Antiphosphotyrosine mouse mAb were purchased from PTM Bio. The IC_50_ value was determined from a sigmoid dose − response curve using Graph-Pad Prism (GraphPad Software, San Diego, CA).

### 2.3. *In vitro* activity assay at cell level

#### 2.3.1. Cell culture

A431 (Human epidermoid carcinoma cell line) cell, A549 (Human non-small cell lung cancer cell line) cell, MCF-7 (Human breast adenocarcinoma cell line) cell, and NCI-H1975 (Human non-small cell lung cancer cell line) cell were purchased from the Shanghai Cell Bank of the Chinese Academy of Sciences. NRK-52E (Normal rat kidney cell line) cell was donated by Guizhou Medical University. All cell lines were maintained in RPMI 1640 or DMEM complete medium.

#### 2.3.2. Cytotoxicity evaluation (MTT assay)

*In vitro* cytotoxicity of compounds **4a–4g** against four cancer cell lines (A431, A549, MCF-7, and NCI-H1975) and normal rat kidney cells (NRK 52E) was determined by MTT assay as our previous report. Gefitinib were used as positive controls. The IC_50_ value was determined from a sigmoid dose − response curve using Graph-Pad Prism (GraphPad Software, San Diego, CA).

#### 2.3.3. Cell apoptosis analysis

The apoptosis of tumour cells MCF-7 treated by different concentrations of compound **4d**, was measured with Annexin V - FITC/PI apoptosis detection kit (Solarbio, Beijing, China), according to instructions of kit, and detected by BD Accuri C6 flow cytometry (American BD Corporation Shanghai Co. Ltd.)

#### 2.3.4. Cell cycle analysis

The distribution of cell cycle for MCF-7 treated by different concentrations of compound **4d**, was measured with Annexin V - FITC/PI cell cycle detection kit (Solarbio, Beijing, China), according to instructions of kit, and detected by BD Accuri C6 flow cytometry (American BD Corporation Shanghai Co. Ltd.)

### 2.4. Molecular docking

X-ray crystal structures of EGFR in both “active” (PDB entry 1M17) and “inactive” (PDB entry 4HJO) states were used for identifying candidate binding modes. The possible binding modes of compound A, compounds **4a–4g**, Gefitinib with EGFR were predicted by molecular docking with Sybyl X-2.0 software from Tripos Inc, St. Louis, MI.

### 2.5. Admet studies

The absorption, distribution, metabolism, elimination, and toxicity (ADMET) parameters of compounds **4a–4g** and Gefitinib were calculated in and calculated in CHARMM Force Field of Discovery Studio 2.5 Software (Accelrys Inc., San Diego, CA). The data of ADMET included Solubility, Absorption, Cytochrome P450 (CYP2D6), Hepatotoxiciy, Plasma protein binding (PPB), Blood brain barrier permeability (BBB), and water partition coefficients for the unionised species (AlogP98)^26^.

## 3. Results and discussions

### 3.1. Chemistry

All target compounds (**4a–4g**) were synthesised and confirmed based on ^1^H-NMR, ^13 ^C-NMR and HRMS. The synthetic route was shown in Scheme 1. At first, compound **2** and **3** were prepared under the described conditions in literature^23^. And then, compound **3** experienced in aldol-condensation with 3-fluorophenylethanone to give the product **4a**. Subsequently, compound **3** reacted with ethyl 4-hydroxybenzoate under the condition of K_2_CO_3_/KI/DMF to give the compound **5** with 64% yield. After hydrolysis in the presence of NaOH, compound **6** was obtained with 58% yield and formed amide **4 b** with 3-fluorobenzenamine. Next, compound **9** was prepared with the reported method and reacted with compound **2** to give styrylquniazolinone **4c**. At last, treatment **2** with 4-nitrophenol and reduce the nitro-group to give the intermediate **11**. Compound **11** reacted with corresponding acid or isocyanate to obtain the final targets **4d–4g**. The synthetic details were described in experimental section and the spectra can be found in the supplementary material.

### 3.2. *In vitro* EGFR kinase inhibitory activity and antitumor activity of target quinazolinone derivatives

With the compounds **4a–4g** in hand, the activity against EGFR^wt^-TK was tested with ELISA assay^27^. As shown in Table 1, when the enamine bond of compound **A** (IC_50_ of 0.047 μM) was substituted with ketene group (Table 1, **4a**), vinyl group (Table 1, **4c**), and amide bond (Table 1, **4b** and **4d**), the IC_50_ values of compounds **4a–d** to EGFR^wt^-TK were 2.71 μM, 0.2 μM, 1.63 μM and 0.053, respectively. Fortunately, compound **4d** reached in the similar activity with compound **A**. Compared to **4d**, 3,4-difluoro substitution on the phenyl ring (Table 1, **4e**) decreased the activity. Meanwhile, the extended amide bond (Table 1, **4f**) and urea bond (Table 1, **4g**) also significantly weaken the activity.

**Table 1.** IC_50_ values for EGFR^wt^-TK. 
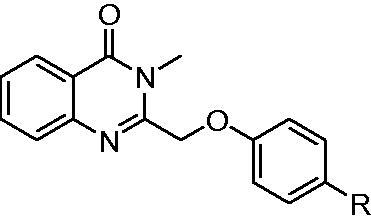

Entry	Comp.	R	EGFR^wt^-TK IC_50_ (μM)
1	**4a**		2.71 ± 0.22
2	**4b**		1.63 ± 0.15
3	**4c**		0.20 ± 0.041
4	**4d**		0.053 ± 0.008
5	**4e**		0.061 ± 0.007
6	**4f**		0.38 ± 0.075
7	**4g**		0.71 ± 0.093
8	**A**		0.047 ± 0.004
**Gefitinib**			0.0061 ± 0.0003

^a^The values are mean ± SD of three replicates.

To investigate the anticancer activity of the synthesised compounds, four EGFR over-expressed human tumour cell lines derived from human epidermoid cancer (A431), non-small cell lung cancer (A549), breast cancer (MCF-7), and non-small cell lung cancer (NCI-H1975) were used to evaluate the antiproliferative activities by methyl thiazolyl tetrazolium colorimetric assay (MTT). Gefitinib, a clinical drug targeted EGFR was employed as positive control. As shown in Table 2, against A431 cells compounds **4d** and **4e** (IC_50_ values of 3.48 μM and 3.53 μM) were more potent than Gefitinib (4.45 μM), while compounds **4a**, **4 b**, **4c**, **4f**, and **4 g** (IC_50_ values of 9.32, 8.72, 6.79, 8.76 μM and 8.34 μM, respectively). Against A549 cells compounds **4c–4f** (with IC_50_ values of 4.26, 2.55, 2.69, 8.46, and 7.31 μM, respectively) were more potent than Gefitinib (8.47 μM). Against NCI-H1975 cells compounds **4c–4f** showed higher inhibitor activities than Gefitinib. Against MCF-7 cells compounds **4d–4f** (with IC_50_ values of 0.87, 0.96, and 2.84 μM, respectively) were more efficient than Gefitinib (5.89 μM).

**Table 2.** IC_50_ values for human cancer cell lines^a^.

Comp.	R	IC_50_ (μM)			
		A431	A549	MCF-7	NCI-H1975
**4a**		9.32 ± 0.57	8.63 ± 0.41	10.58 ± 1.03	13.68 ± 1.61
**4b**		8.72 ± 0.46	9.08 ± 0.69	9.64 ± 0.88	12.87 ± 1.44
**4c**		6.79 ± 0.72	4.26 ± 0.57	6.04 ± 0.73	6.44 ± 0.85
**4d**		3.48 ± 0.49	2.55 ± 0.26	0.87 ± 0.14	6.42 ± 0.47
**4e**		3.53 ± 0.34	2.69 ± 0.32	0.96 ± 0.21	7.18 ± 0.49
**4f**		8.76 ± 0.74	8.46 ± 0.67	2.84 ± 0.22	9.33 ± 0.78
**4g**		8.35 ± 0.46	7.31 ± 0.59	10.74 ± 2.07	10.19 ± 1.03
**A**		17.71 ± 2.08	17.86 ± 2.11	14.09 ± 1.85	19.18 ± 3.02
**Gefitinib**		4.45 ± 0.25	8.47 ± 1.06	5.89 ± 0.72	9.71 ± 1.96

^a^The values are mean ± SD of three replicates.

Based on the result of EGFR^wt^-TK and four tumour cell assays, we found that the similar structure–activity relationship (SAR) was observed in cell lines and EGFR-TKs. The amide group (**4d** and **4e**) instead of enamine was more potent than the ketene group (**4a**), vinyl group (**4c**), urea group (**4 g**) and other groups (**4 b** and **4f**). Besides, compound **A** showed lower inhibitor activities against four tumour cell lines than the compounds **4a–4g**. The reason may be due to the lower stability of compound **A** in cells. Generally, compound **4d** was the best in the synthesised compounds and particularly showed much higher inhibitor activities than Gefitinib against four tumour cells. These data indicate that **4d** was worth to further investigation.

### 3.3. *In vitro* cytotoxicity of 3-methyl quinazolinone derivatives on normal cells

**Figure 2.**
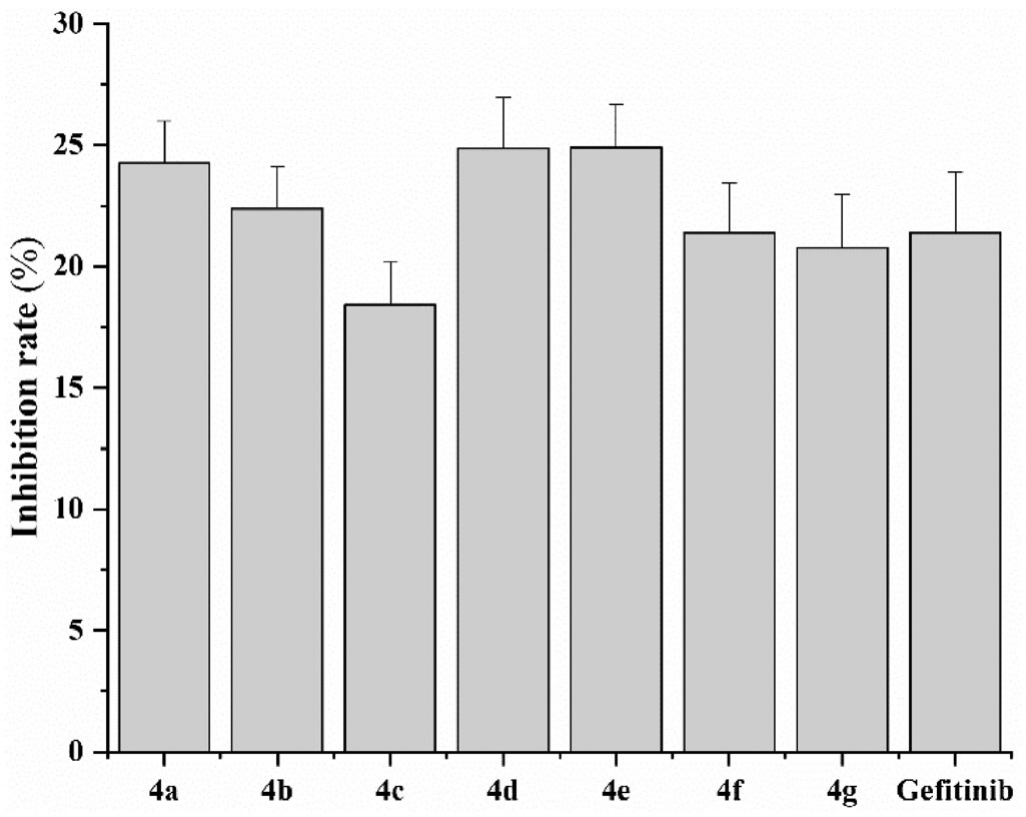
*In vitro* cytotoxicity of compounds **4a-g** and **Gefitinib** on NRK-52E cell.

The cytotoxicity study of compounds **4a–4g** was first evaluated by MTT colorimetric assays to normal rat kidney cell line (NRK-52E) at different concentrations. There was no inhibition of all compounds including Gefitinib at 10 μM. Therefore, the concentration was accelerated to 100 μM. As shown in Figure 2, all compounds were similar with Gefitinib and not more than 25% against NRK-52E at 100 μM. This indicate that the synthesised compounds **4a–4g** were low cytotoxicity and potent for development for drugs.

### 3.4. Effects of compound 4d on cell apoptosis of MCF-7 cell line

**Figure 3.**
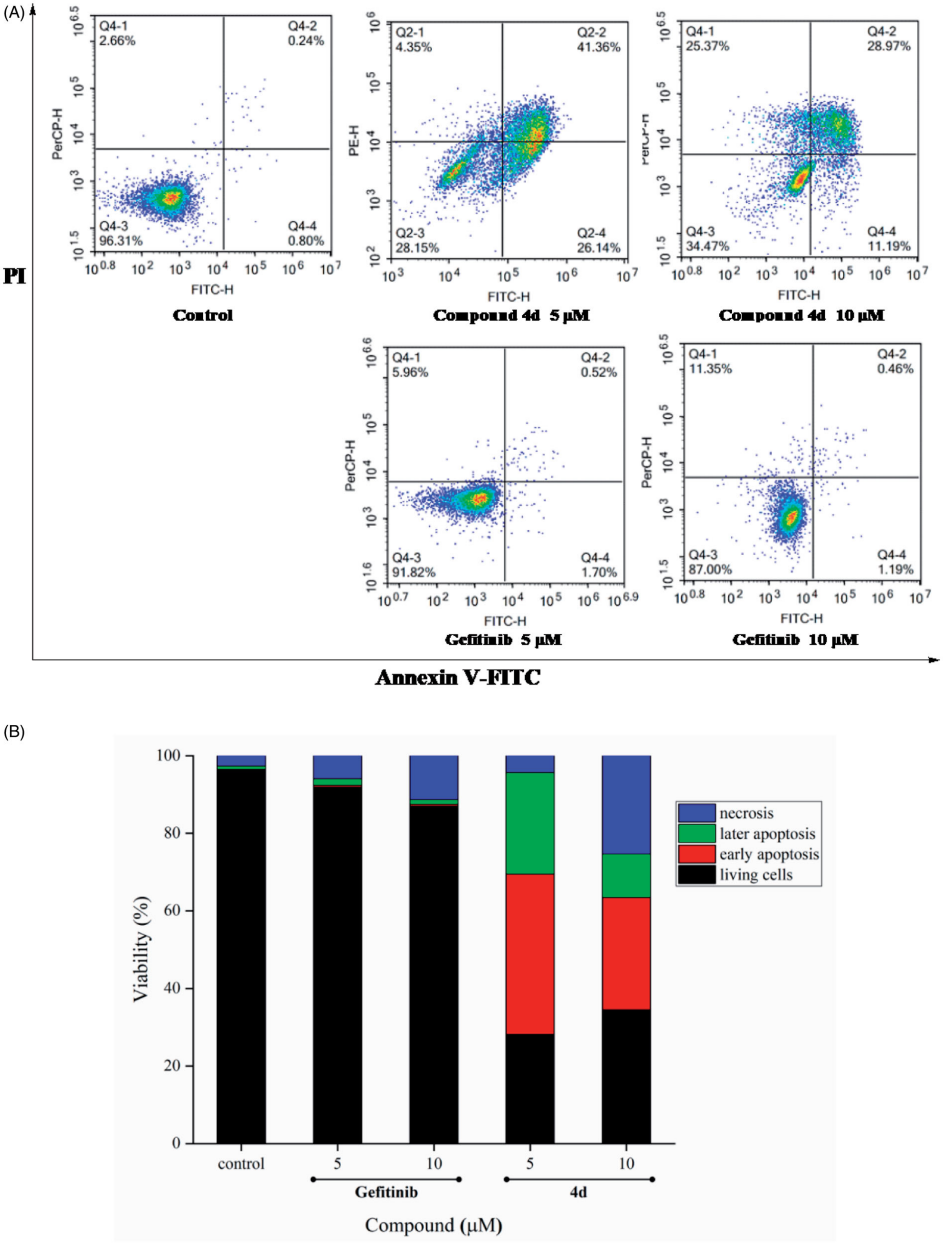
Compound **4d** induced cell apoptosis in Annexin V-FITC assay. (A) Density plot were obtained by flow cytometry, Gefitinib was used as reference drug. (B) Total apoptotic cells (%) at various concentrations of **4d** and Gefitinib.

Apoptosis of tumour cell is the basic mechanism of tissue homeostasis, which is regarded as the efficient method to eliminate the excess cells^28^. Most of the anti-cancer drugs perform as a well inducing apoptosis for cancer cells^29^. As shown in Figure 3, compound **4d** was used to investigate the mechanism of inhibition of MCF-7 cell proliferation. Flow cytometry analysis of MCF-7 treated with **4d** at 5 and 10 µM for 48 h demonstrated a prominent increase in apoptotic cells with a dose-dependent fashion. The trend of compound **4d** showed that early apoptosis rate was priority over late apoptosis. Compared compound **4d** with Gefitinib, the results in Figure 3(B) showed that **4d** significantly performed better ability to induce early apoptosis at the same concentration. Particularly, the ratio of apoptotic cells for compound **4d** reached 28.14% (early) and 41.36% (late) even at 5 µM.

### 3.5. Effects of compound 4d on cell cycle of MCF-7 cell line

**Figure 4.**
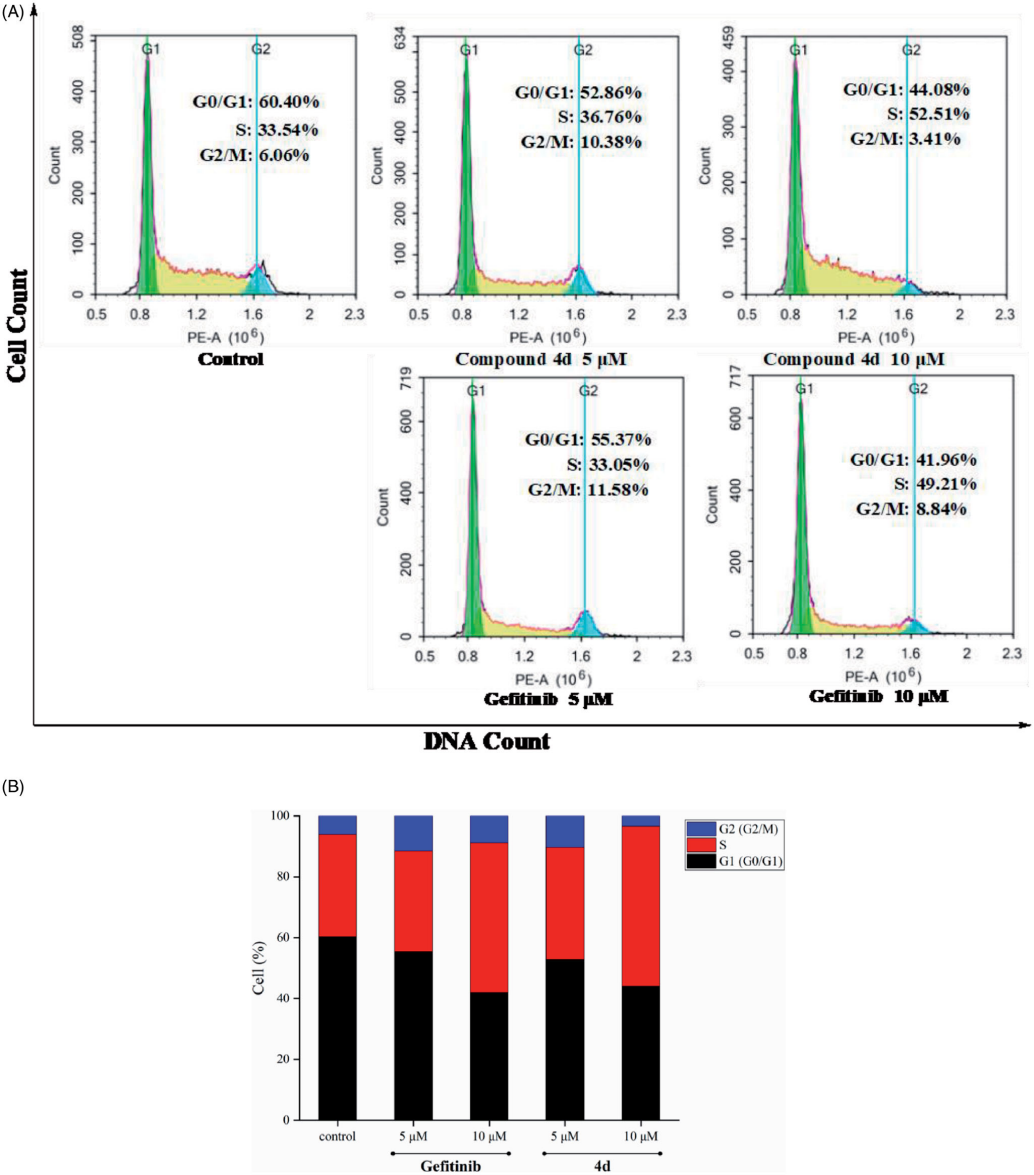
Cell cycle distribution of compound **4d** and Gefitinib against MCF-7 was studied by flow cytometry. (A) Profiles were obtained by FACS. The percentages for different phases of cell cycle were illustrated in the histogram. (B) MCF-7 cells were cultured in the presence of different concentrations of **4d** (5 μM and 10 μM) or Gefitinib (5 μM and 10 μM) for 48 h, harvested, fixed, and labelled with PI, then analysed by FACS. Percentage of cells in G0/G1, S and G2/M phases are indicated.

Next, we wished to determine whether **4d**-induced decrease of EGFR phosphorylation would result in cell cycle arrest in MCF-7 cell lines, compared with Gefitinib. As shown in Figure 4, cell cycle analysis was tested in flow cytometry. MCF-7 cell lines were treated with compound **4d** at 5 and 10 µM. Against MCF-7 cells at 5 µM, the control consisted of 6.06% G2 phase cells, compound **4d** increased to 10.38% and Gefitinib also reached to 11.58%. However, at 10 μM of tested samples, G2/M phase decreased and S phase increased, this might be that compound **4d** and Gefitinib undergo significant apoptosis. These results indicate that compound **4d** and Gefitinib could arrest MCF-7 cells in G2/M phase at the appropriate concentration.

### 3.6. Molecular docking studies

To explain the structural activity relationship of EGFR with our compounds, the possible binding modes were performed in molecular docking studies^30^. Former researches showed that the “active” or “inactive” state of EGFR-TK has different preference of ligand binding profile, so X-ray crystal structures of EGFR in both “active” (PDB entry 1M17) and “inactive” (PDB entry 4HJO) states were used for identifying candidate binding modes^31^.

**Figure 5.**
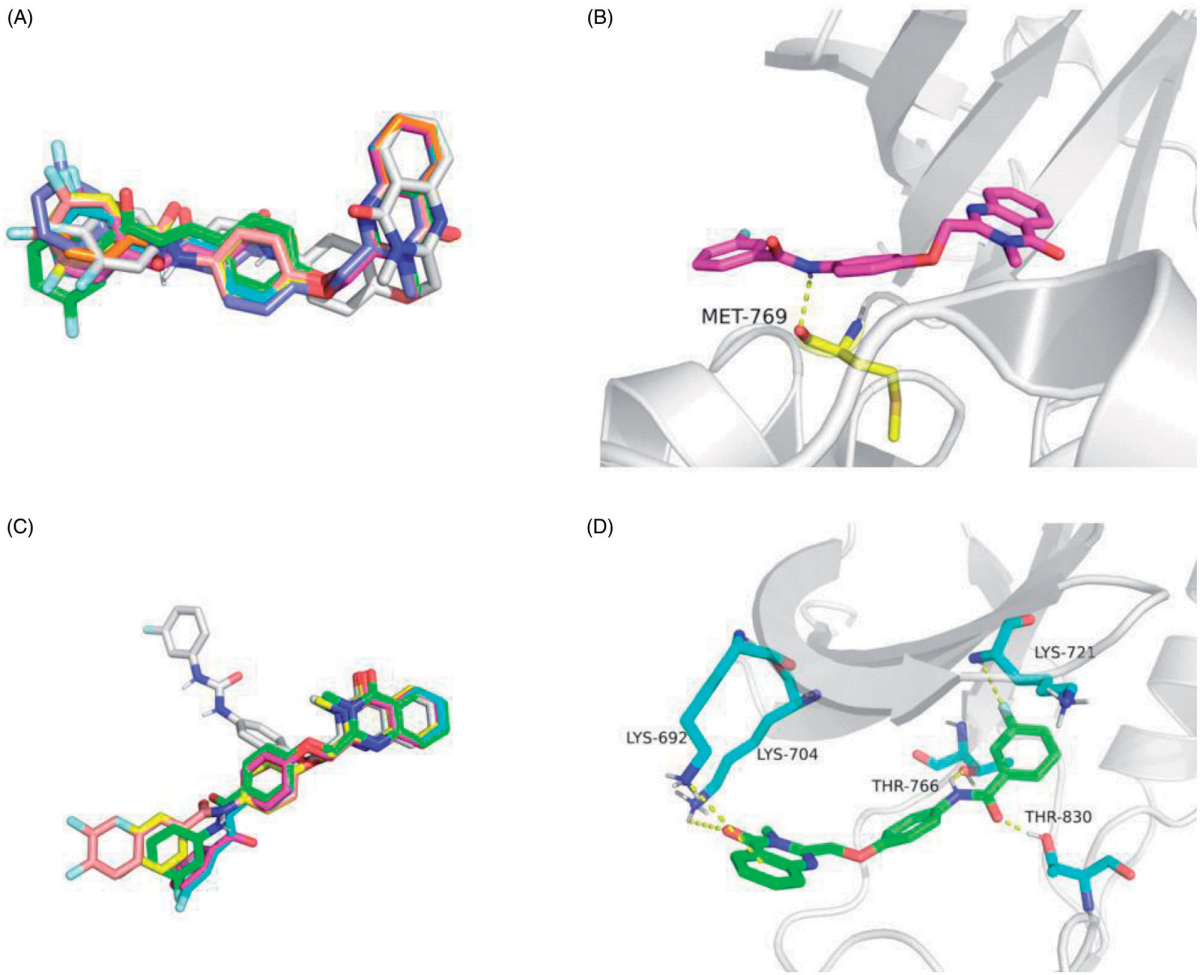
Docking structures of compounds **4a-4g**. (A) Different binding configurations of compounds **4a-4g** with EGFR (PDB: 1M17). (B) The 3 D model of compound **4d** bound to EGFR (PDB: 1M17). (C) Different binding configurations of compounds **4a-4g** with EGFR (PDB: 4HJO). (D) The 3 D model of compound **4d** bound to EGFR (PDB: 4HJO).

As shown in Tables S1 and S2, the predicted score of all compounds **4a–4g** with inactive state of EGFR (PDB: 4HJO) is much higher than the score of active state of EGFR (PDB: 1M7), which suggests that the compounds **4a–4g** are likely to bind with EGFR in inactive state. By contrast, the predicted binding model for Gefitinib in two states are similar, which is consistent with our previous work. More interestingly, as shown in Figure 5(A,C), all the compounds **4a–4g** were employed in the same binding configuration with EGFR active state or inactive state. Take compound **4d** as example, in Figure 5(B) for active state, simply formed one hydrogen bond with Met769 during the docking situation. However, in Figure 5(D) for inactive state, quinazolinone ring of compound **4d** formed stable hydrogen bonds with Lys692 and Lys704. Meanwhile, amide group and fluorine atom of **4d** also formed hydrogen bonds with Thr766, Thr830, and Lys721 respectively. These results indicate that the synthesised compounds **4a–4g** are particularly likely to be potential EGFR inhibitors against inactive state.

### 3.7. Admet and stability studies

The ADMET are essential aspects for drug candidates^32^. Computer aided ADMET studies have been employed in the early stage of drug discovery. Therefore, the ADMET properties of compounds **4a–4g** were calculated through Discovery Studio 2.5 software (Accelrys Inc., San Diego, CA). As shown in Table 3, the levels of solubility and absorption were the same with compounds **4a–4g** and Gefitinib. CYP2D6 is non-inhibitor of cytochrome P450 enzyme for predicting drug toxicity. The compounds **4a**, **4f** and **4 g** were less toxic than Gefitinib, and the others were similar with Gefitinib. Meanwhile, the calculated log *p* values of compound **4 b**, **4d**, **4e** and **4 g** were less than Gefitinib (4.20). Particularly, compound **4d** reached the lowest log p (3.52) and performed highly oral bioavailability. These data are consistent with the structure-activity relationships.

**Table 3.** ADMET properties of compounds **4a–g**.

Comp.	Solubility	Absorption	CYP2D6	Hepatotoxicity	PPB	BBB	AlogP98
**4a**	2	0	0	1	2	1	4.61
**4b**	2	0	1	1	2	2	3.53
**4c**	2	0	1	1	2	1	4.73
**4d**	2	0	1	1	2	2	3.52
**4e**	2	0	1	1	2	2	3.73
**4f**	2	0	0	1	2	1	4.20
**4g**	2	0	0	1	2	2	3.63
**Gefitinib**	2	0	1	0	1	1	4.20

At last, we investigated the stability of compound **4d** in mice plasma. The results exhibited that compound **4d** was stable with purity higher than 98% after incubation with Sprague Dawley (SD) mice plasma (Figure S2). All the data suggest that compound **4d** was a good candidate for developing new anti-cancer drug.

## 4. Conclusions

To solve the issues of metabolic stability in our previous research of EGFR inhibitors, seven 3-methylquinazolinone derivatives were designed and synthesised. After evaluated activities of these compounds in enzyme and cell level, we found that compounds **4d** and **4e** were performed well antiproliferative activities. Furthermore, the best compound **4d** displayed IC_50_ value of 53 nM against EGFR^wt^-TK activity and IC_50_ value of 0.87 μM against MCF-7 cells. Analysis of apoptosis and cell cycle for MCF-7 cells indicate that compound **4d** could induce apoptosis and arrest in G2/M phase at tested concentration. At last, molecular docking, ADMET, and stability studies suggested that compound **4d** was closely formed hydrogen bonds with EGFR^wt^-TK and potential to develop new anti-cancer drug.

## Supplementary Material

Supplemental Material
