# Supplementary material for: Synthesis and preliminary structure-activity relationship study of 3-methylquinazolinone derivatives as EGFR inhibitors with enhanced antiproliferative activities against tumour cells
Source: J Enzyme Inhib Med Chem. 2021 Jun 2;36(1):1205–16. doi: 10.1080/14756366.2021.1933466 (PMC8174486; doi:10.1080/14756366.2021.1933466)
Supplement: Supplemental Material [file IENZ_A_1933466_SM8390.pdf]

Supporting Information

Synthesis and structure-activity relationship study of 3-methylquinazolinone derivatives as EGFR inhibitors with enhanced antiproliferative activities against tumor cells

Yan Zhang [a,b,c], Qin Wang [a,b,c], Luolan Li [b,d], Yi Le [a,b,c,*], Li Liu [a,c], Jing Yang [a], Yongliang Li [e,*], Guochen Bao [f], and Longjia Yan [a,b,c,*]

[a] School of Pharmaceutical Sciences, Guizhou University, Guiyang 550025, China

[b] State Key Laboratory of Functions and Applications of Medicinal Plants, Guizhou Medical University, Guiyang 550014, China

[c] Guizhou Engineering Laboratory for Synthetic Drugs, Guiyang 550025, China

[d] Shizhen College of Guizhou University of Traditional Chinese Medicine, Guiyang 550025, China

[e] Faculty of Light Industry and Chemical Engineering, Guangdong University of Technology, Guangzhou, 510006, China

[f] Institute for Biomedical Materials and Devices (IBMD), Faculty of Science, University of Technology Sydney, Sydney, New South Wales, Australia

Corresponding Author: yile2021@163.com (Yi Le), yongliangli@gdut.edu.cn (Yongliang Li), ylj1089@163.com (Longjia Yan).

Table of contents

Docking Score………… ……………………………………………………………………..…..**2**

HPLC procedures and spectra in plasma ….…..….………….……………………..…..**3**

NMR and HRMS spectra ………………………………………………………………..…..**4-23**

Table S1. Docking score of **4a-4g**, **Gefitinib** with EGFR (PDB: 1M17)

Name	Total Score	Crash	Polar	Similarity	D Score	PMF Score	G Score	Chem Score
**4a**	8.7995	-1.3863	0.0001	0.458	-156.313	4.5079	-244.522	-31.089
**4b**	8.3296	-1.0948	0.0256	0.464	-155.251	-18.615	-249.388	-27.9545
**4c**	8.8895	-0.9629	0.0001	0.417	-148.999	-18.8676	-233.61	-30.4704
**4d**	8.6704	-1.224	0.0041	0.466	-150.186	-14.8862	-238.508	-28.3862
**4e**	8.6655	-1.2402	0.0026	0.457	-156.399	-19.4138	-239.602	-28.1691
**4f**	10.1183	-3.1831	0.8078	0.486	-187.671	0.5441	-286.714	-32.2659
**4g**	8.8794	-1.4078	1.5936	0.410	-153.563	-33.0871	-234.828	-34.5688
**Gefitinib**	12.3998	-2.4315	2.1795	0.390	-169.596	-8.459	-287.137	-26.9932

Table S2. Docking score of **4a-4g**, **Gefitinib** with EGFR (PDB: 4JHO)

Name	Total Score	Crash	Polar	Similarity	D Score	PMF Score	G Score	Chem Score
**4a**	10.0280	-0.7298	1.2546	0.520	-141.4576	-7.0184	-228.2086	-29.4758
**4b**	10.8192	-0.6655	2.3815	0.438	-144.8873	-8.1774	-232.1974	-32.8916
**4c**	11.3993	-0.4236	1.2573	0.430	-136.9127	-24.0616	-224.4798	-33.2973
**4d**	10.6816	-0.6028	1.2325	0.463	-144.0467	-26.4526	-218.4806	-30.3043
**4e**	10.3793	-0.8659	2.2913	0.469	-141.4165	-1.0862	-222.6039	-32.2469
**4f**	10.9124	-0.9148	2.4753	0.458	-152.9281	-6.8209	-252.4425	-34.4713
**4g**	10.5807	-0.7372	2.2703	0.398	-141.0358	-31.3597	-257.5684	-31.4444
**Gefitinib**	11.2510	-3.4342	1.7535	0.411	-185.5575	-6.5145	-326.7711	-31.7016

HPLC procedures and spectra

A 2.5 mL centrifuge tube was added 300 μL plasma derived from normal Sprague Dawley (SD) mice (7 weeks, male), and 30 μL compound **A** or compound **4d** in ethanol (50 mM). The mixture was incubated at 37 °C for 60 min, and then treated with 300 μL of CH_3CN, separated in centrifugation at 30000 rpm for 10 min. The supernatant was treated with the same procedure for another time and analyzed in HPLC using C18 column (4.6 mm x 250 mm) and mobile phase of CH_3CN/H_2O (90 : 10) with a flow rate of 1.5 mL/min.

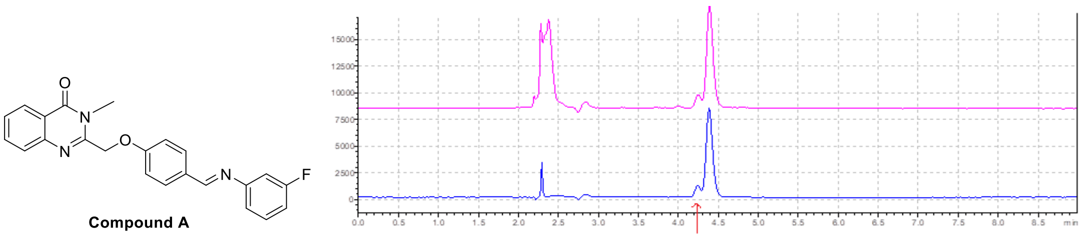

Fig. S1 Metabolic stability in plasma of compound **A**

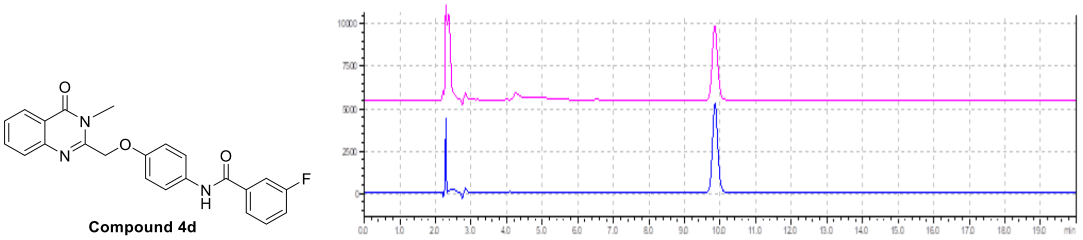

Fig. S2 Metabolic stability in plasma of compound **4d**

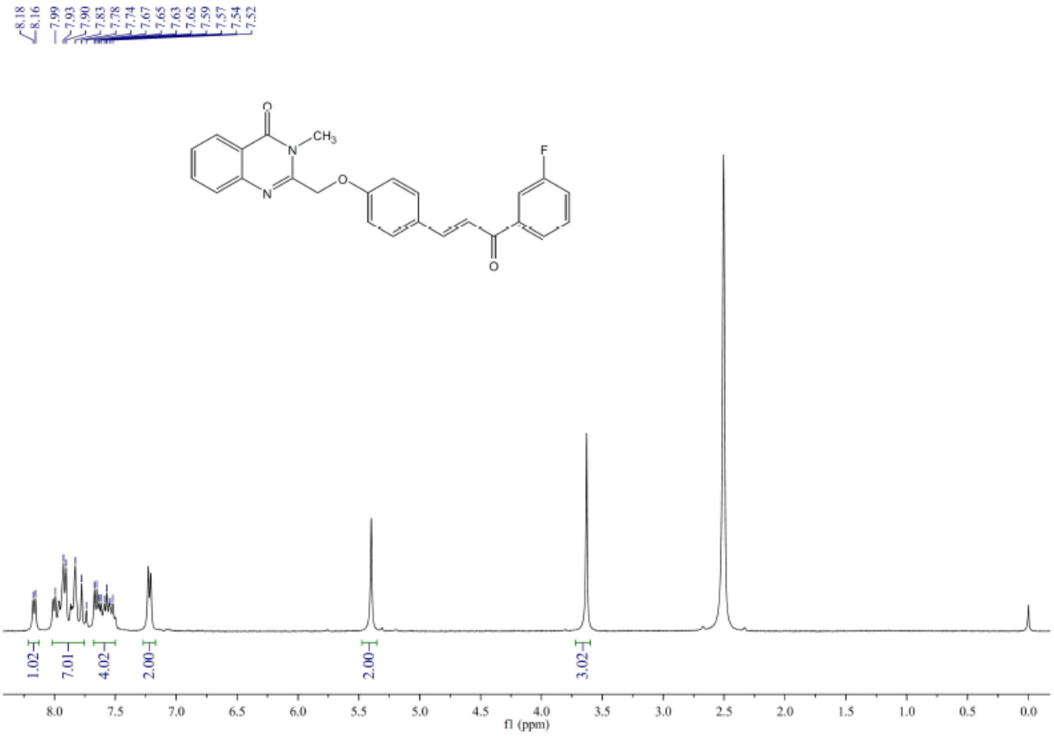

Fig. S3 ^{1}H NMR spectrogram (400 MHz, DMSO) of compound **4a**

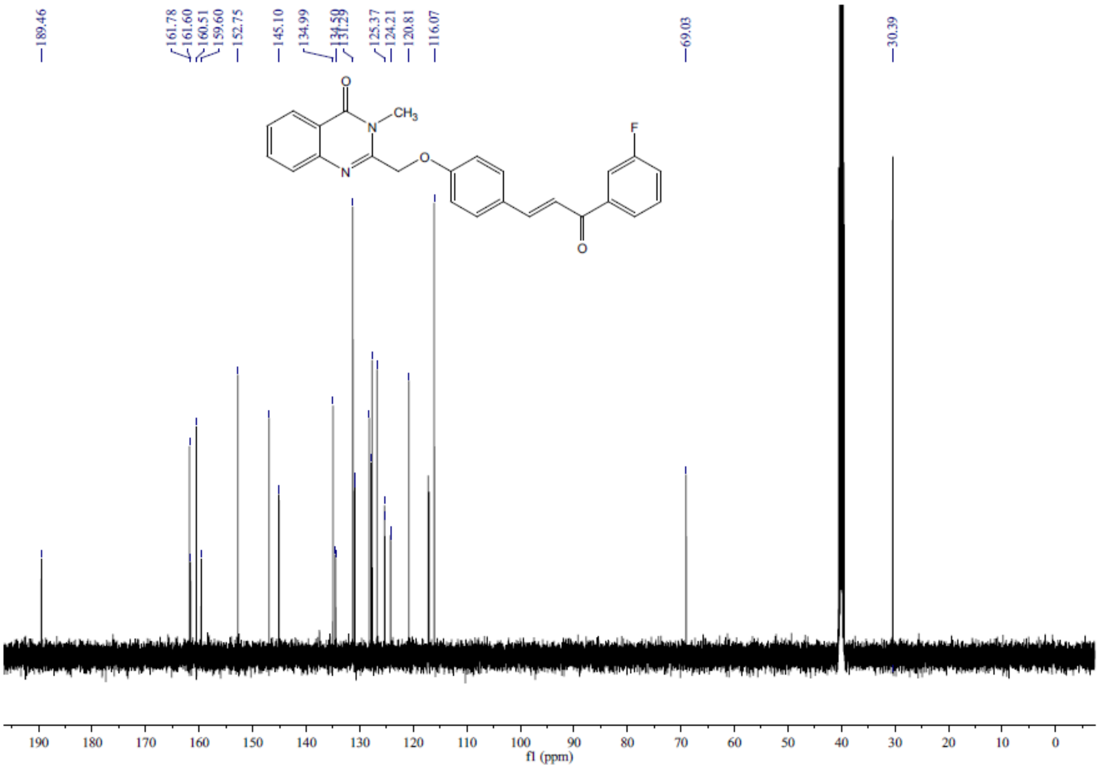

Fig.S4 ^{13}C NMR spectrogram (125 MHz, DMSO) of compound **4a**

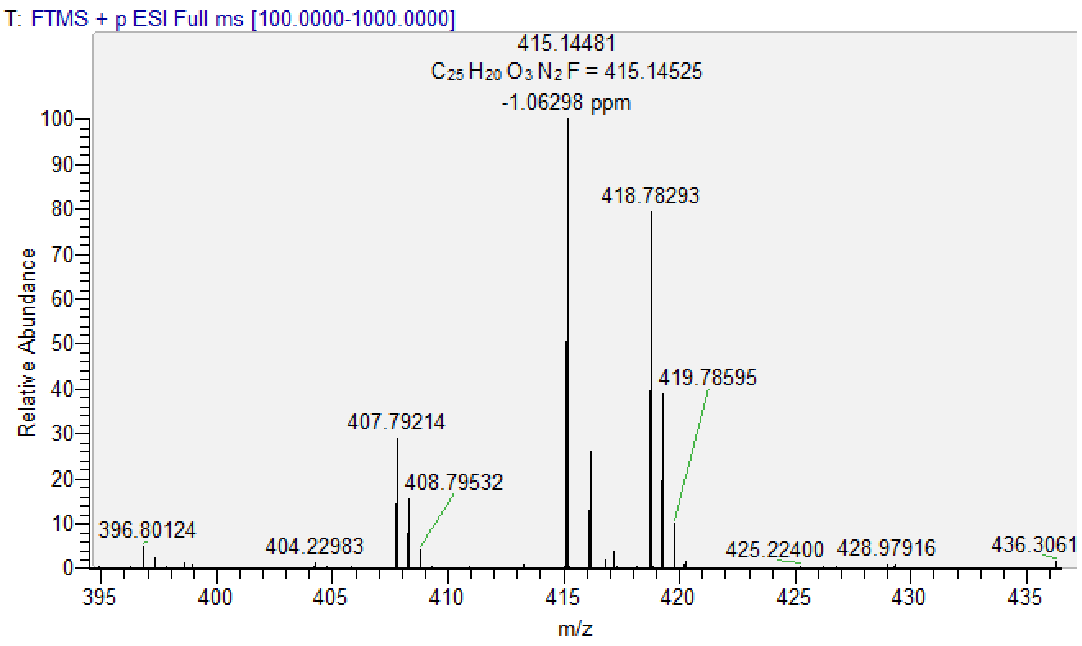

Fig.S5 HR-MS (ESI) spectrogram of compound **4a**

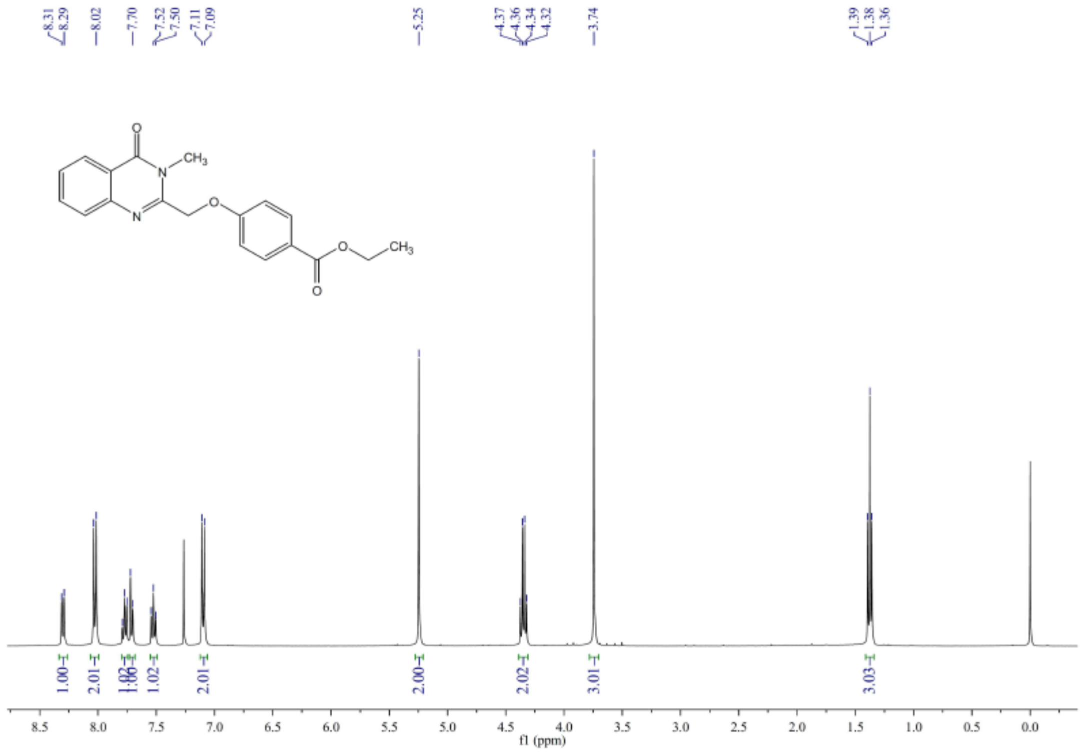

Fig.S6 ^{1}H NMR spectrogram (400 MHz, $CDCl_3$) of compound **5**

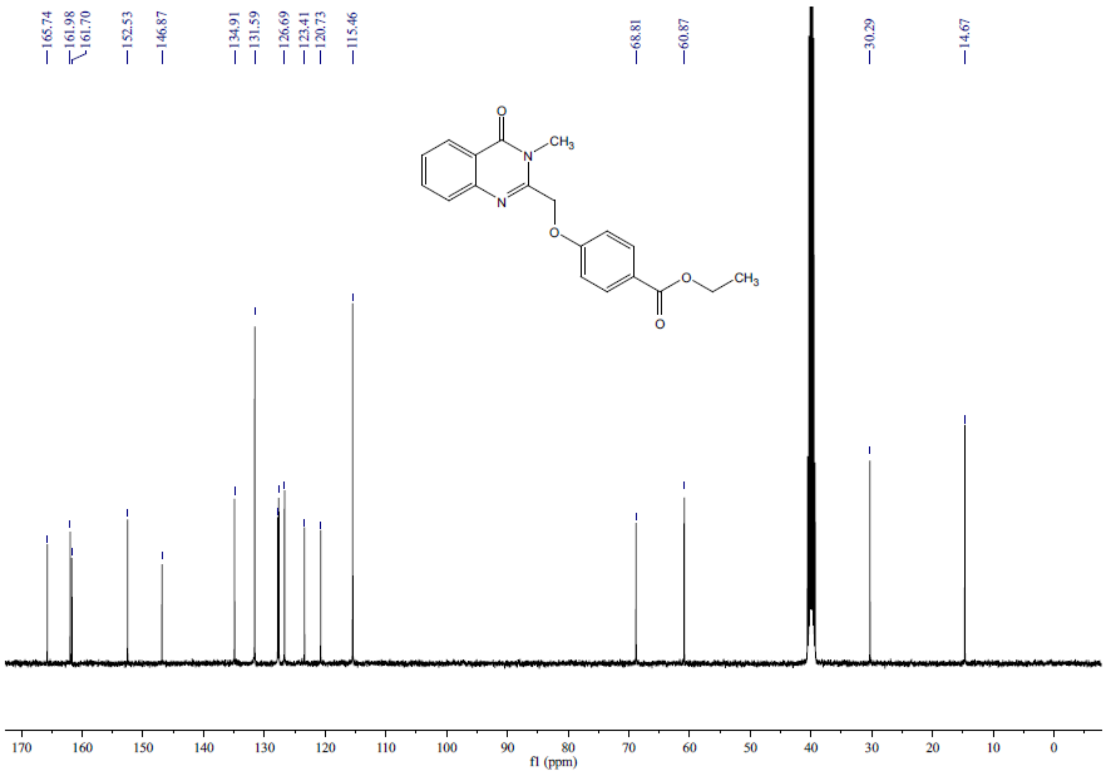

Fig.S7 ^{13}C NMR spectrogram (100 MHz, DMSO) of compound **5**

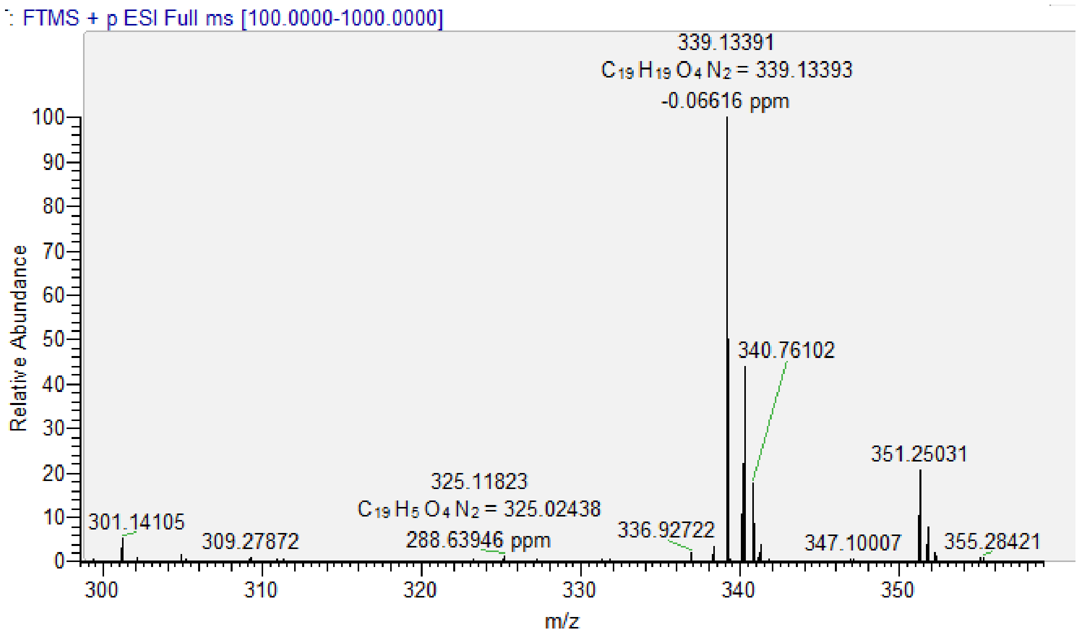

Fig.S8 HR-MS (ESI) spectrogram of compound **5**

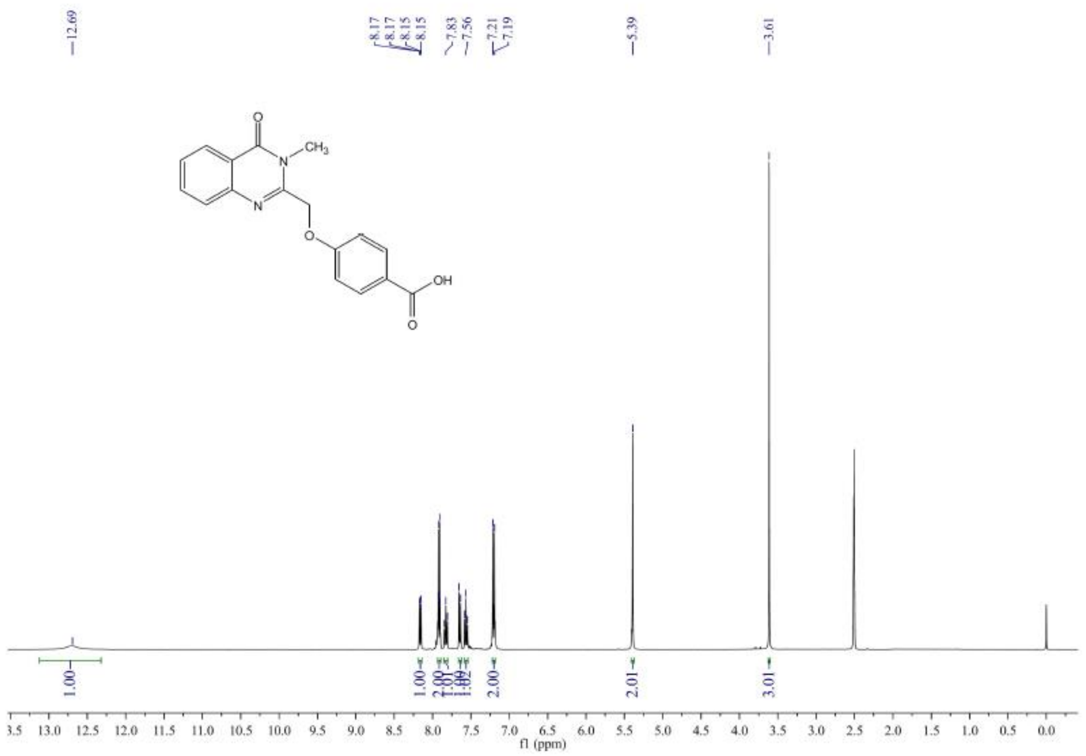

Fig.S9 ^{1}H NMR spectrogram (400 MHz, DMSO) of compound **6**

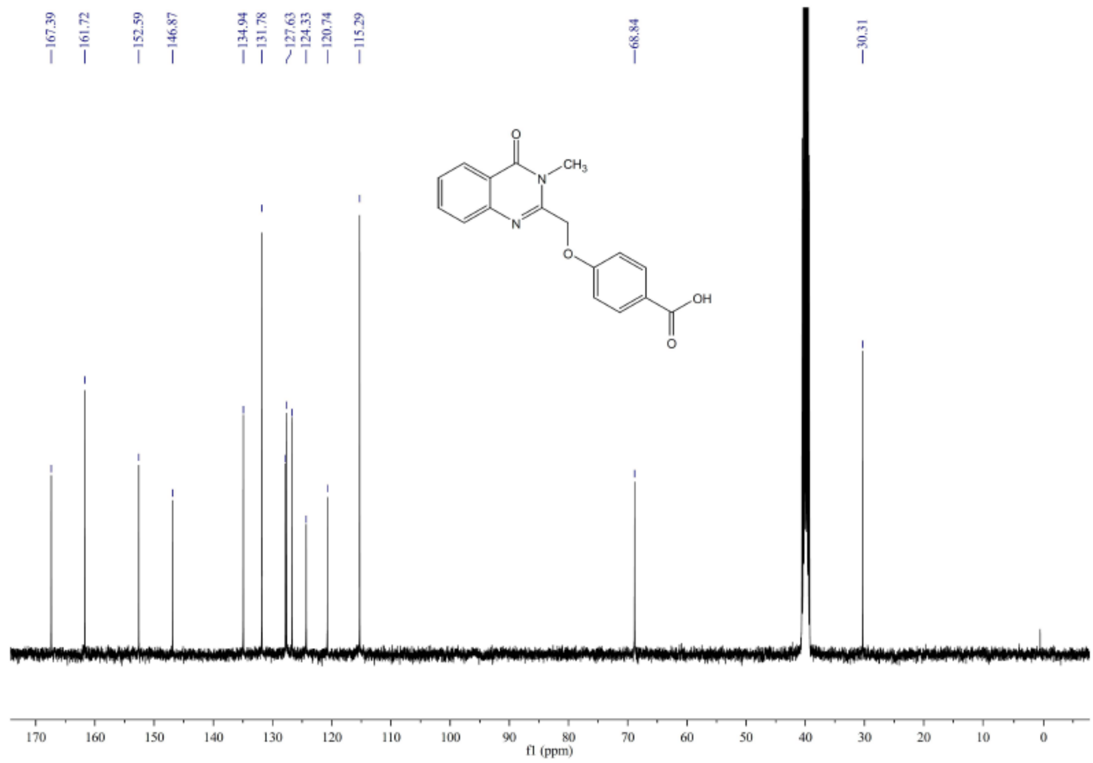

Fig.S10 ^{13}C NMR spectrogram (100 MHz, DMSO) of compound **6**

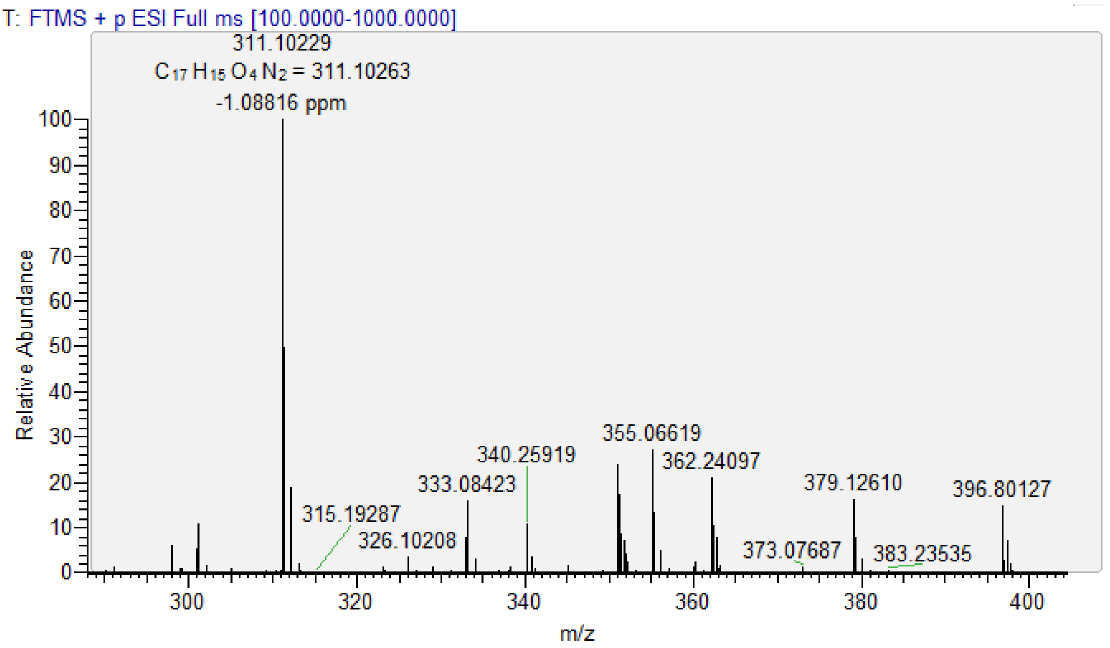

Fig.S11 HR-MS (ESI) spectrogram of compound **6**

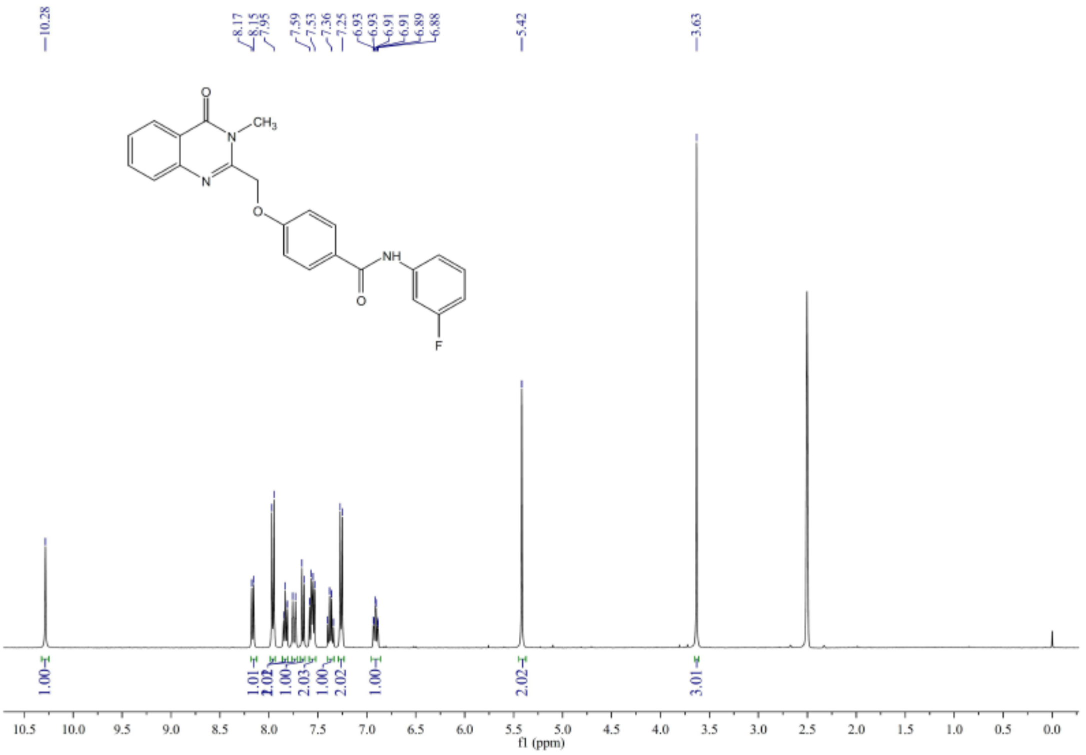

Fig.S12 ^{1}H NMR spectrogram (400 MHz, DMSO) of compound **4b**

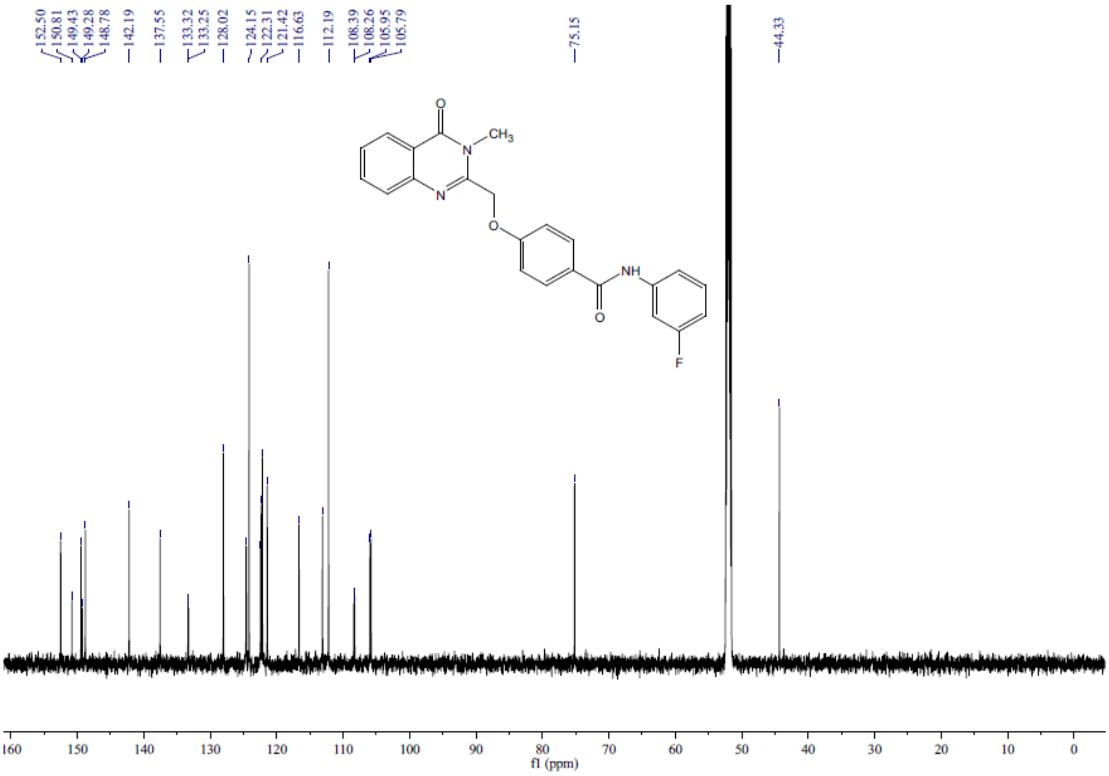

Fig.S13 ^{13}C NMR spectrogram (125 MHz, DMSO) of compound **4b**

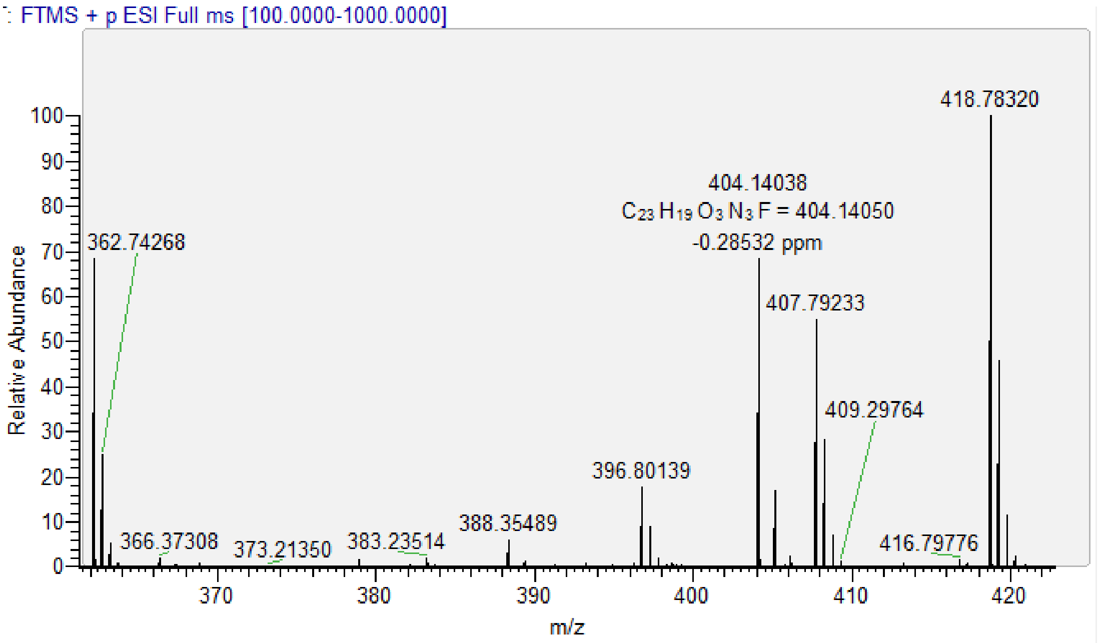

Fig.S14 HR-MS (ESI) spectrogram of compound **4b**

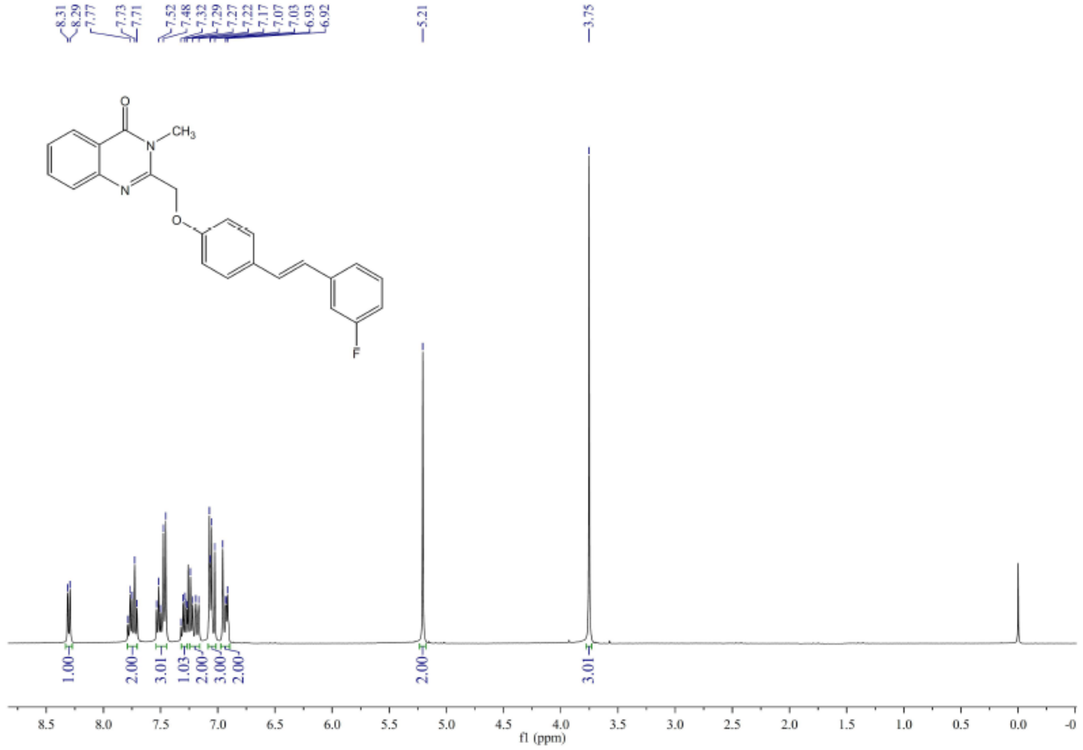

Fig.S15 ^{1}H NMR spectrogram (400 MHz, $CDCl_3$) of compound **4c**

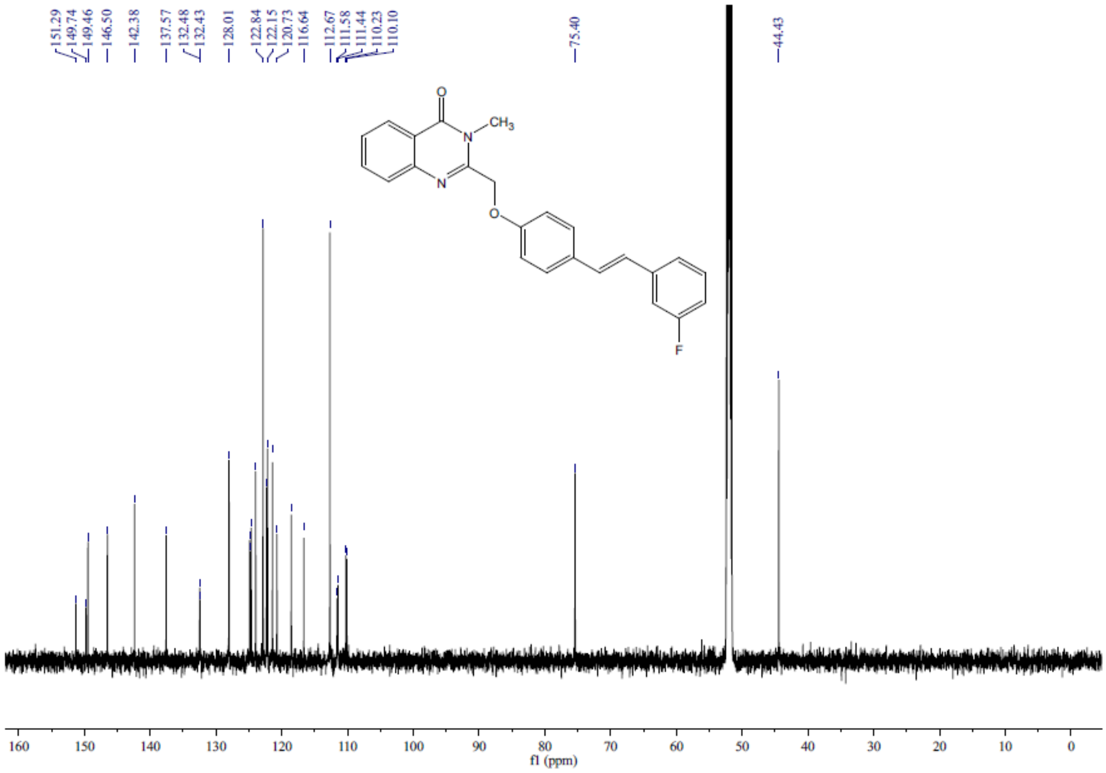

Fig.S16 ^{13}C NMR spectrogram (125 MHz, DMSO) of compound **4c**

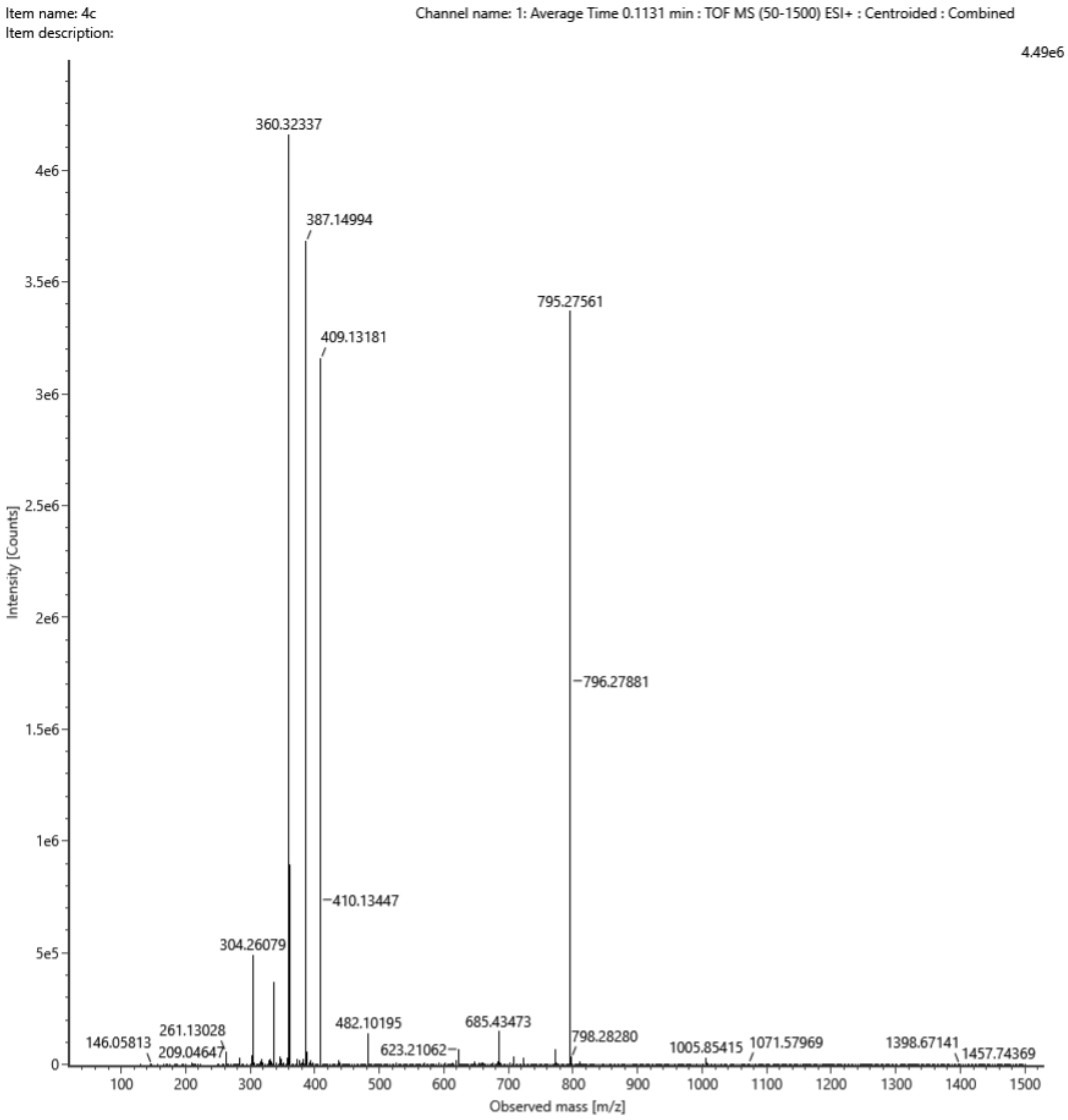

Fig.S17 HR-MS (ESI) spectrogram of compound **4c**

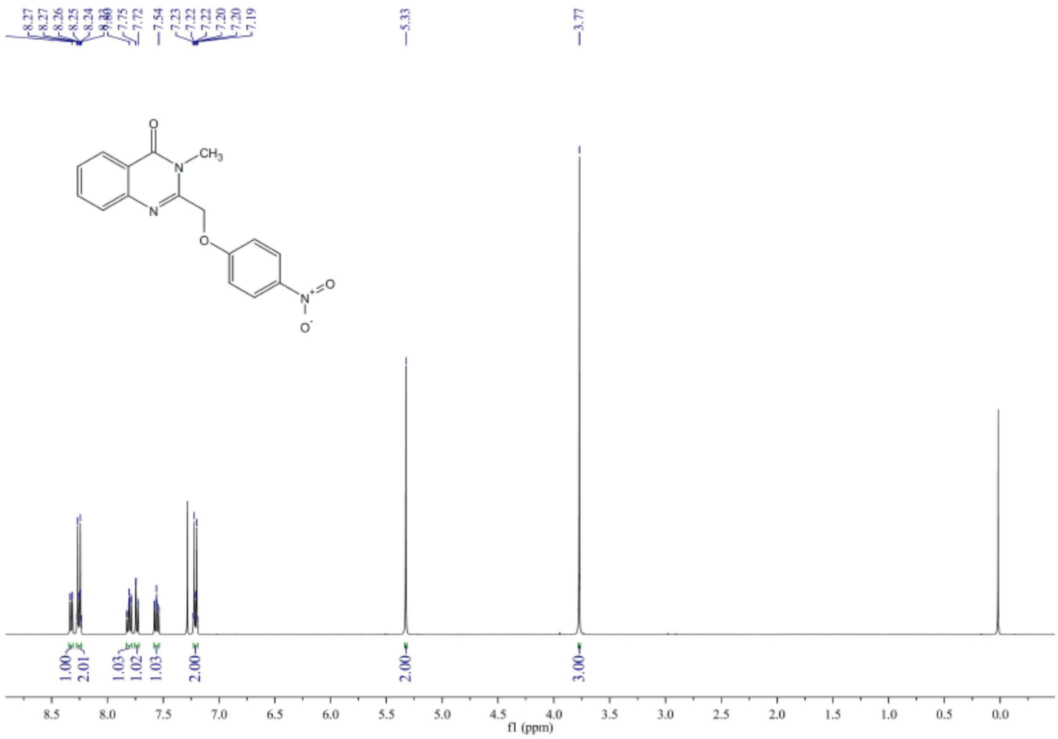

Fig.S18 ^{1}H NMR spectrogram (400 MHz, $CDCl_3$) of compound **10**

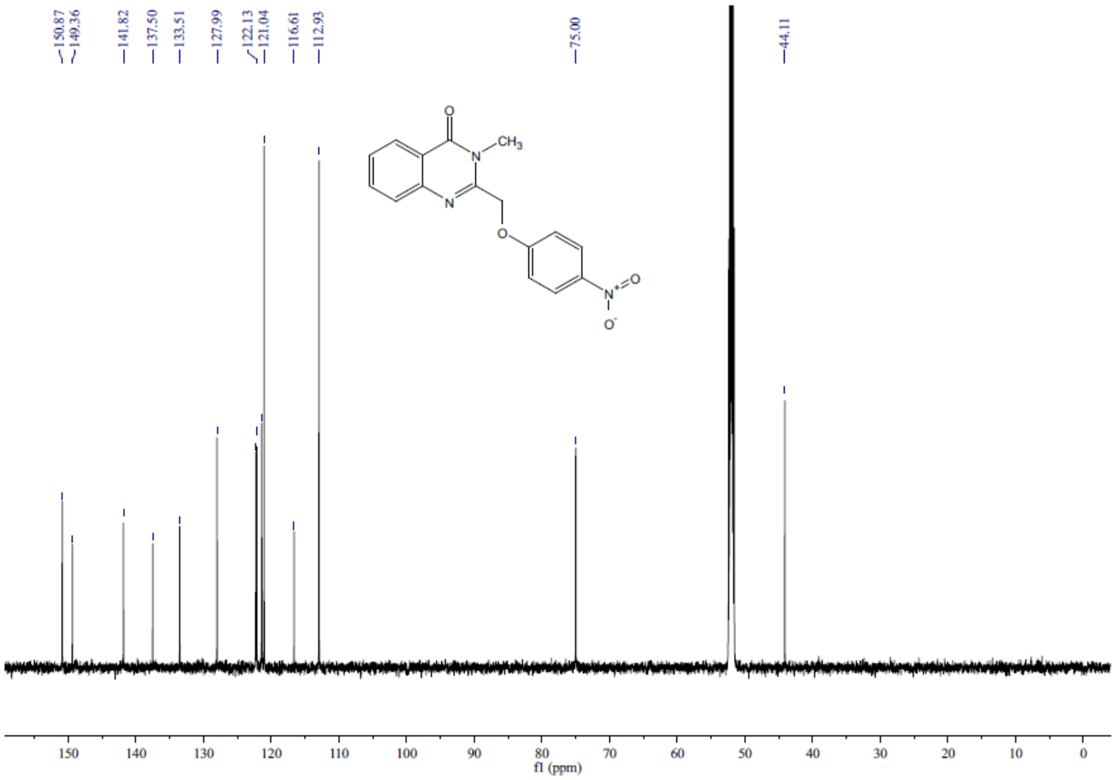

Fig.S19 ^{13}C NMR spectrogram (125 MHz, DMSO) of compound **10**

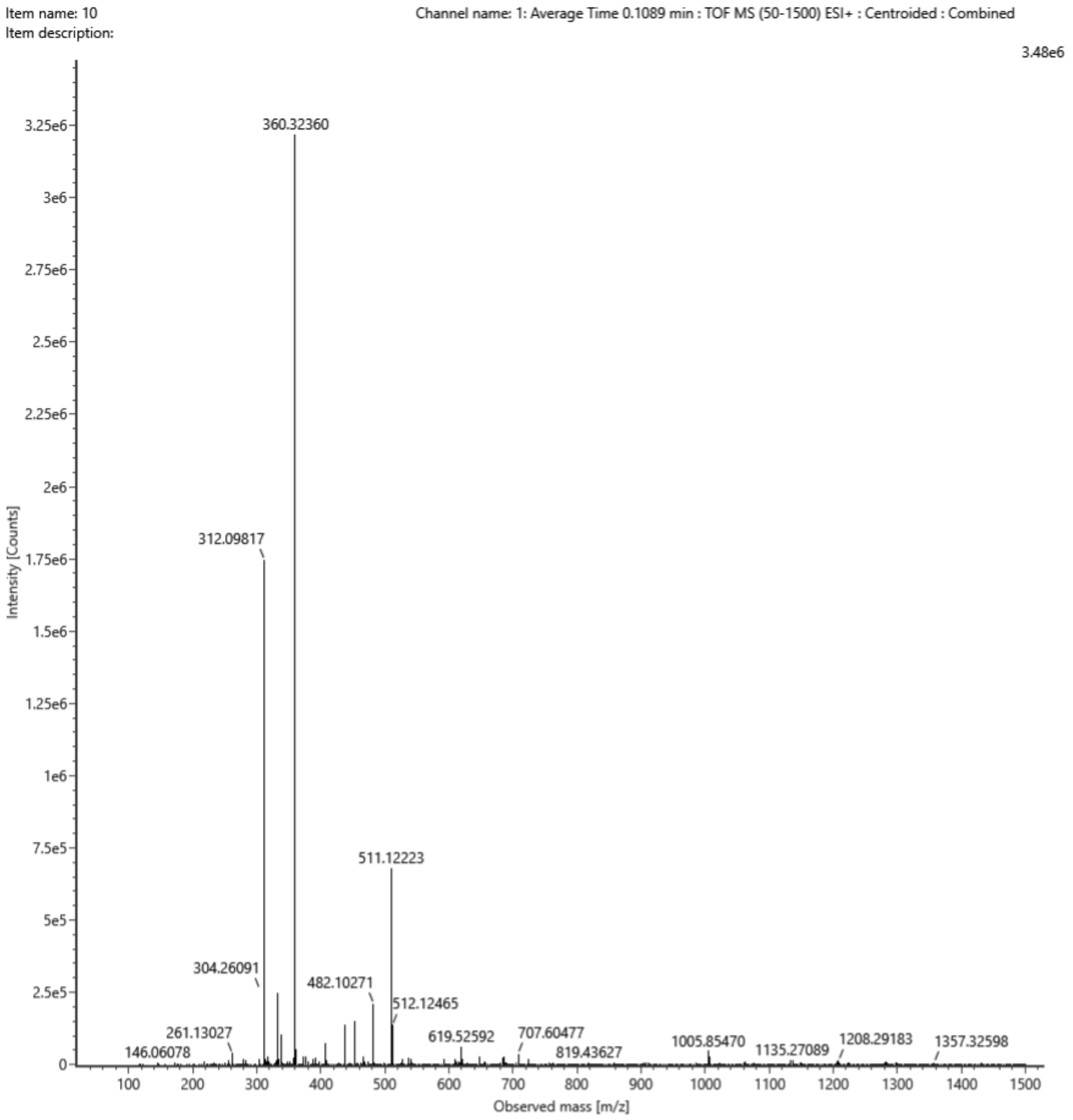

Fig.S20 HR-MS (ESI) spectrogram of compound **10**

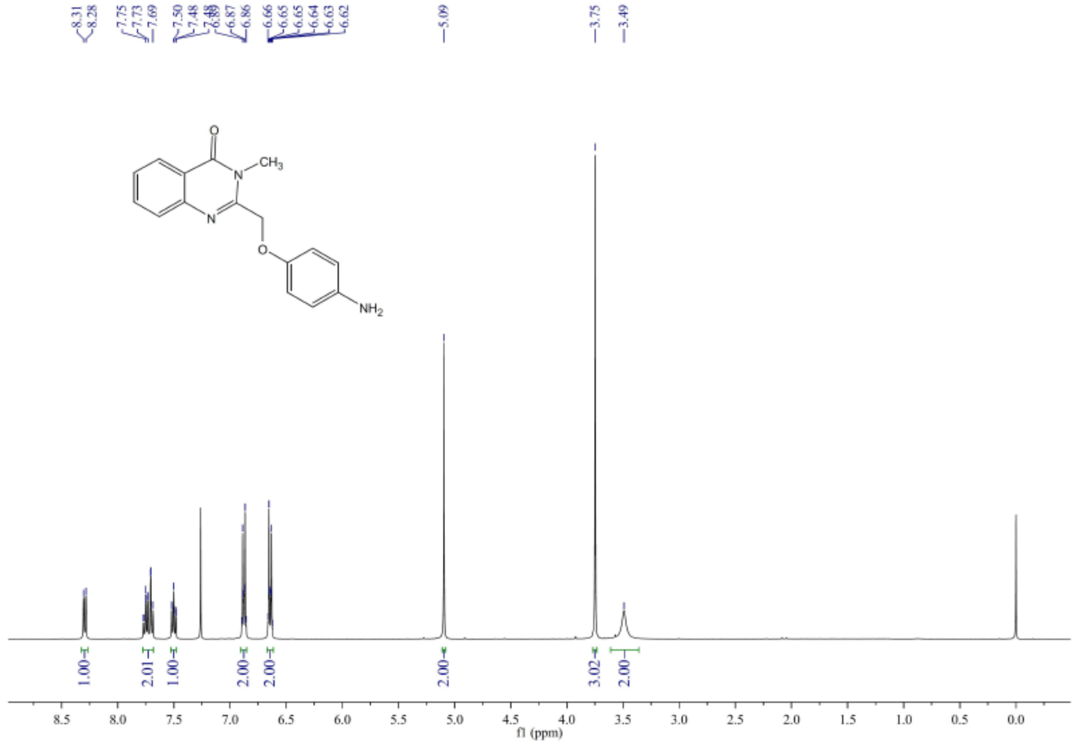

Fig.S21 ^{1}H NMR spectrogram (400 MHz, $CDCl_3$) of compound **11**

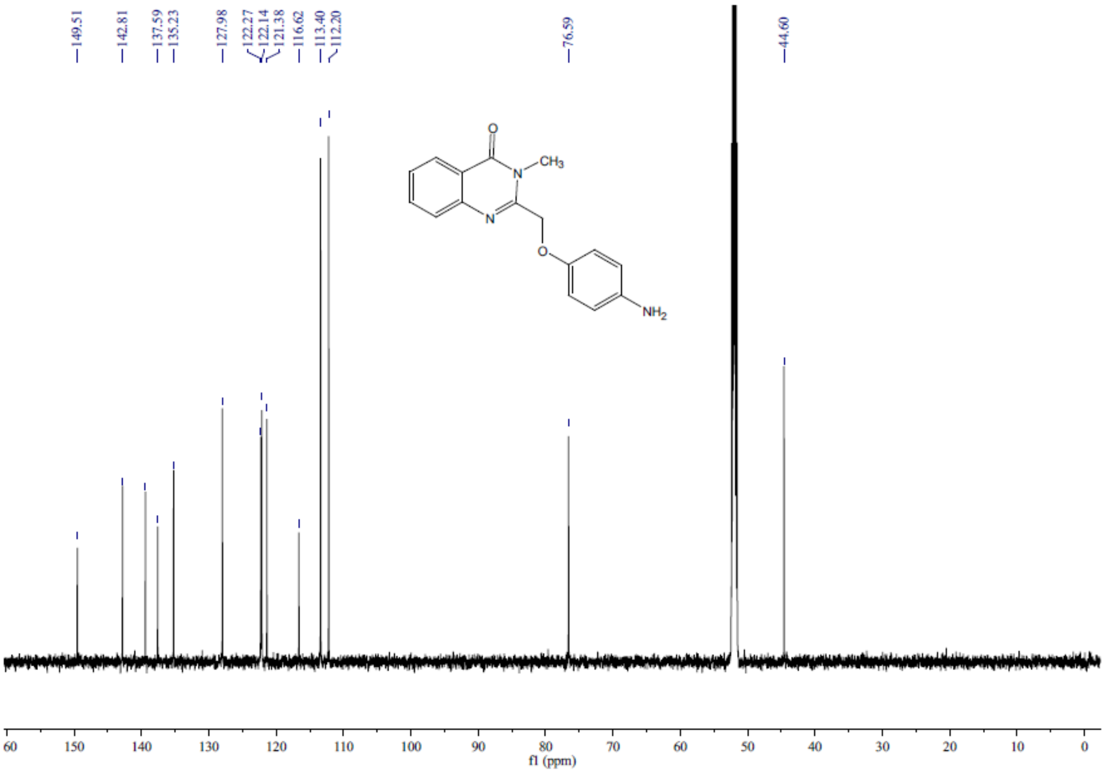

Fig.S22 ^{13}C NMR spectrogram (125 MHz, DMSO) of compound **11**

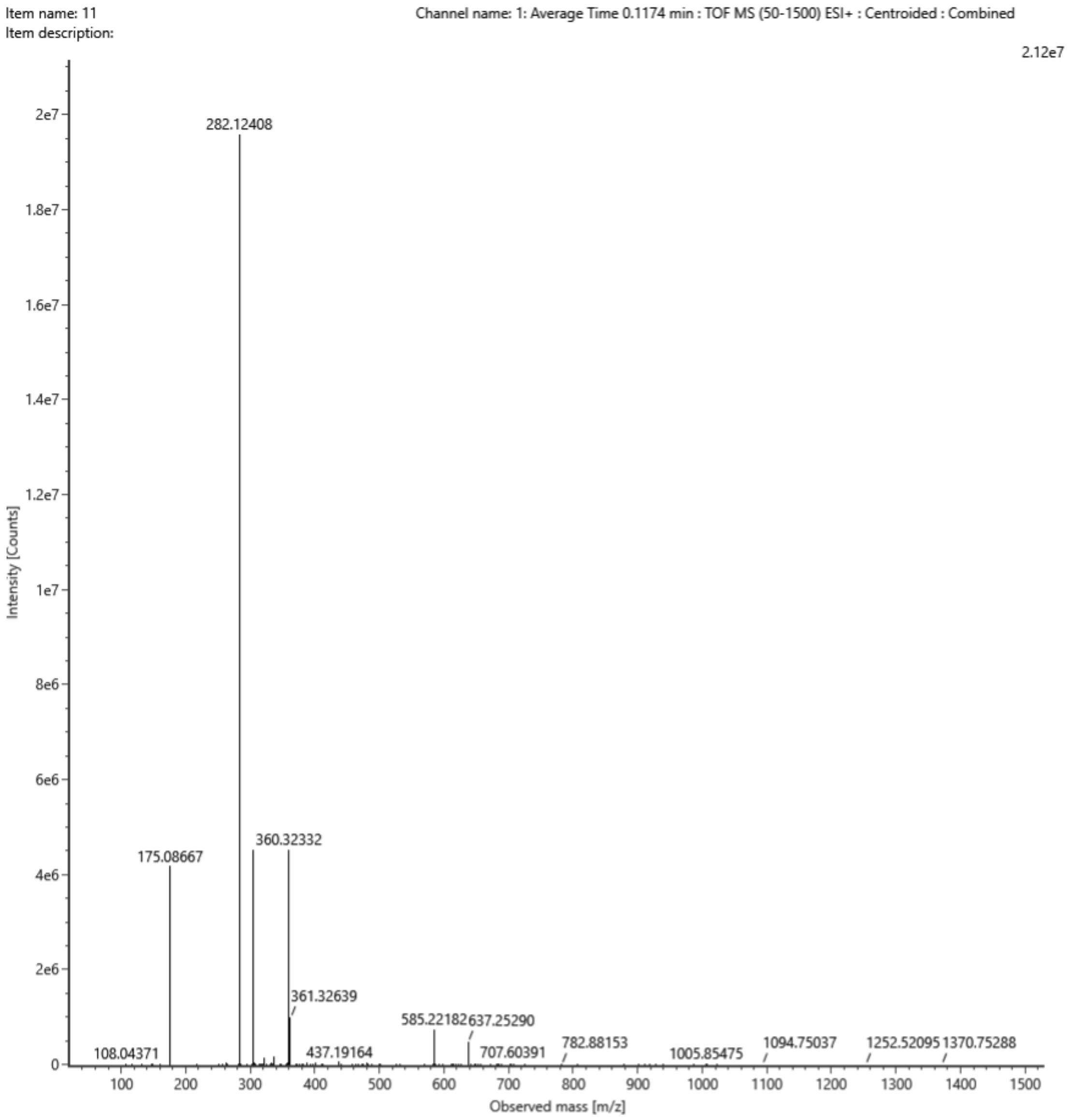

Fig.S23 HR-MS (ESI) spectrogram of compound **11**

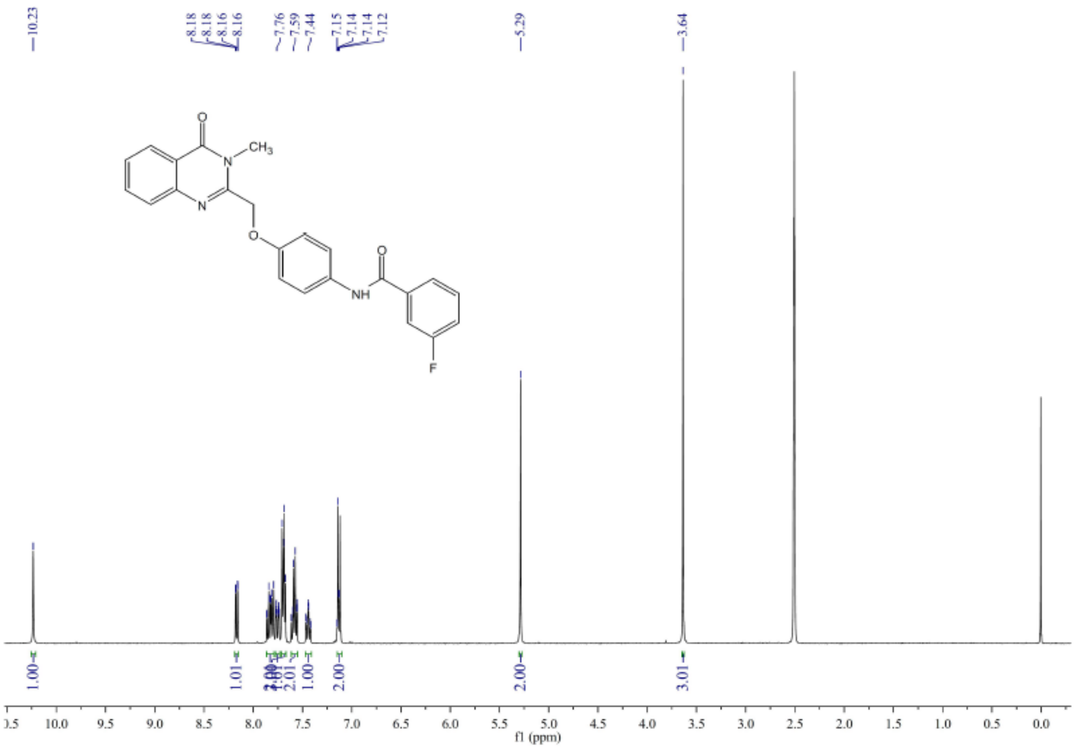

Fig.S24 ^{1}H NMR spectrogram (400 MHz, DMSO) of compound **4d**

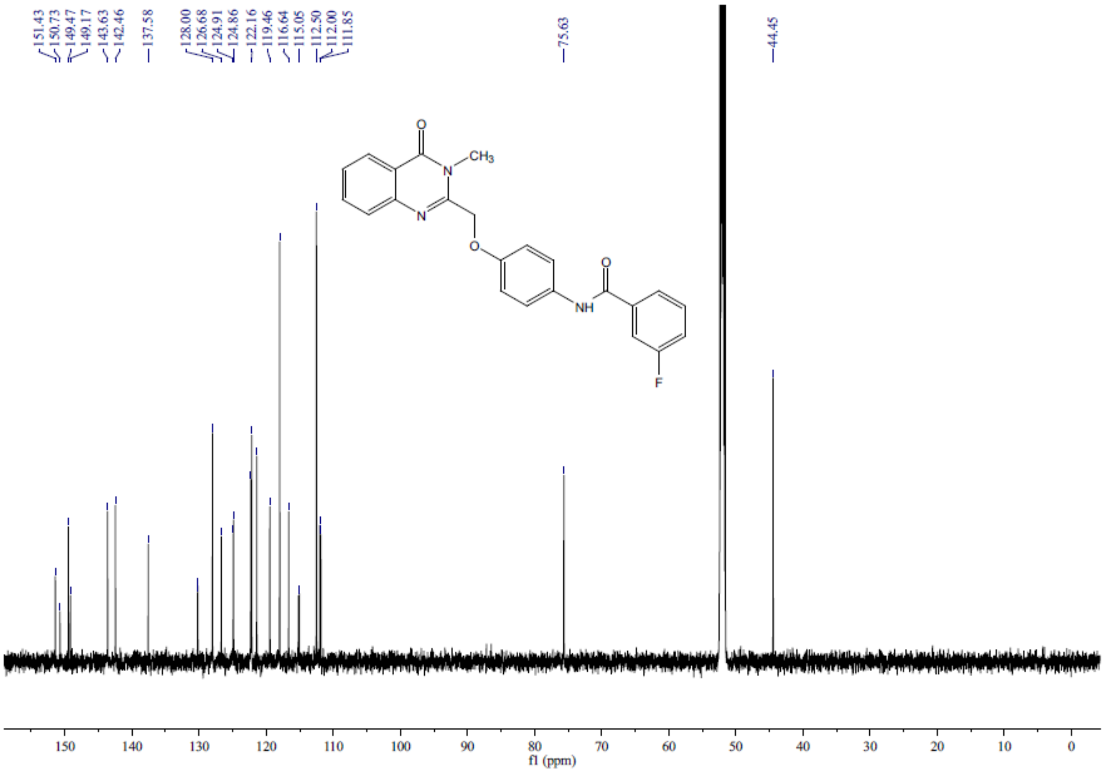

Fig.S25 ^{13}C NMR spectrogram (125 MHz, DMSO) of compound **4d**

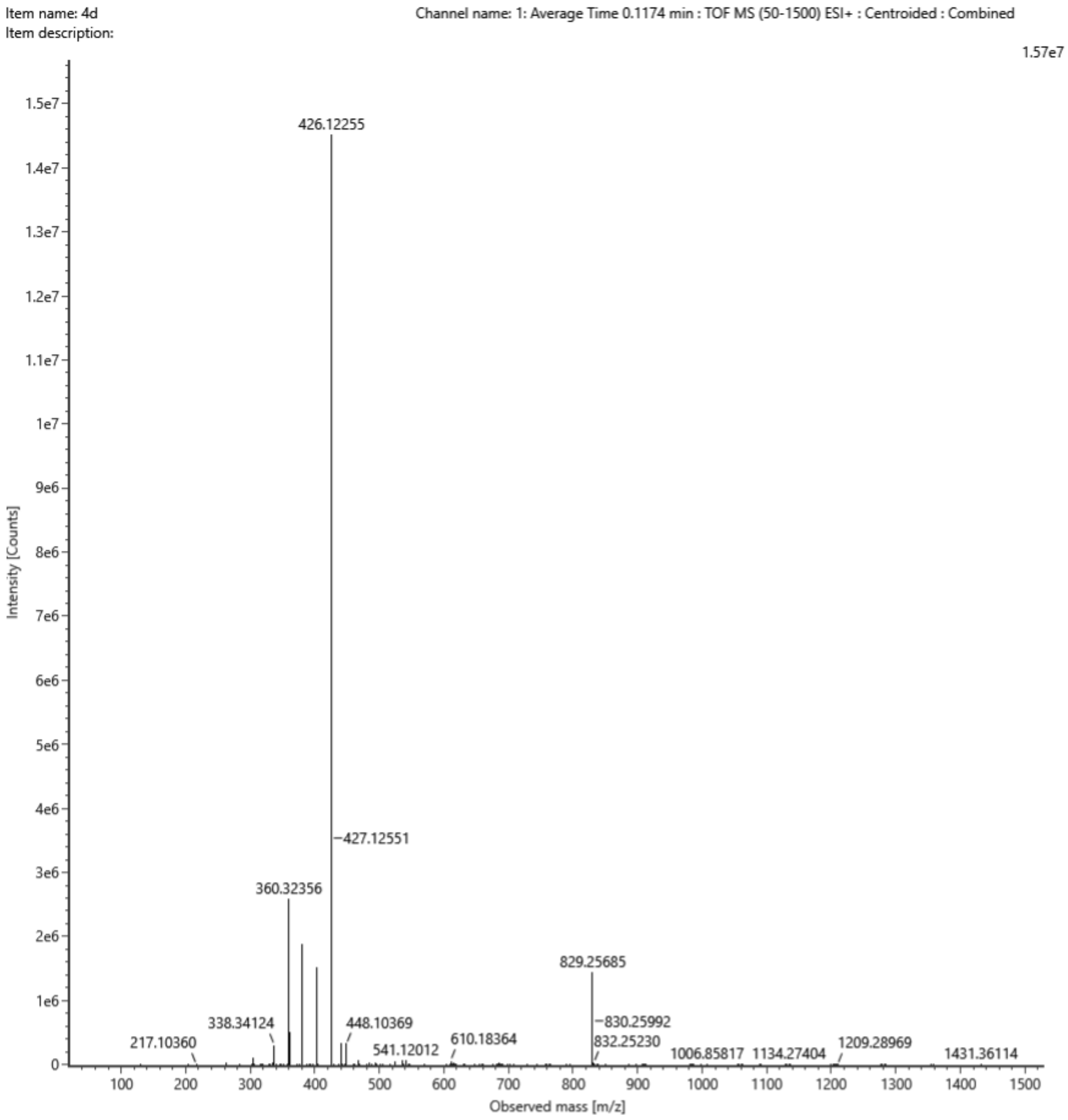

Fig.S26 HR-MS (ESI) spectrogram of compound **4d**

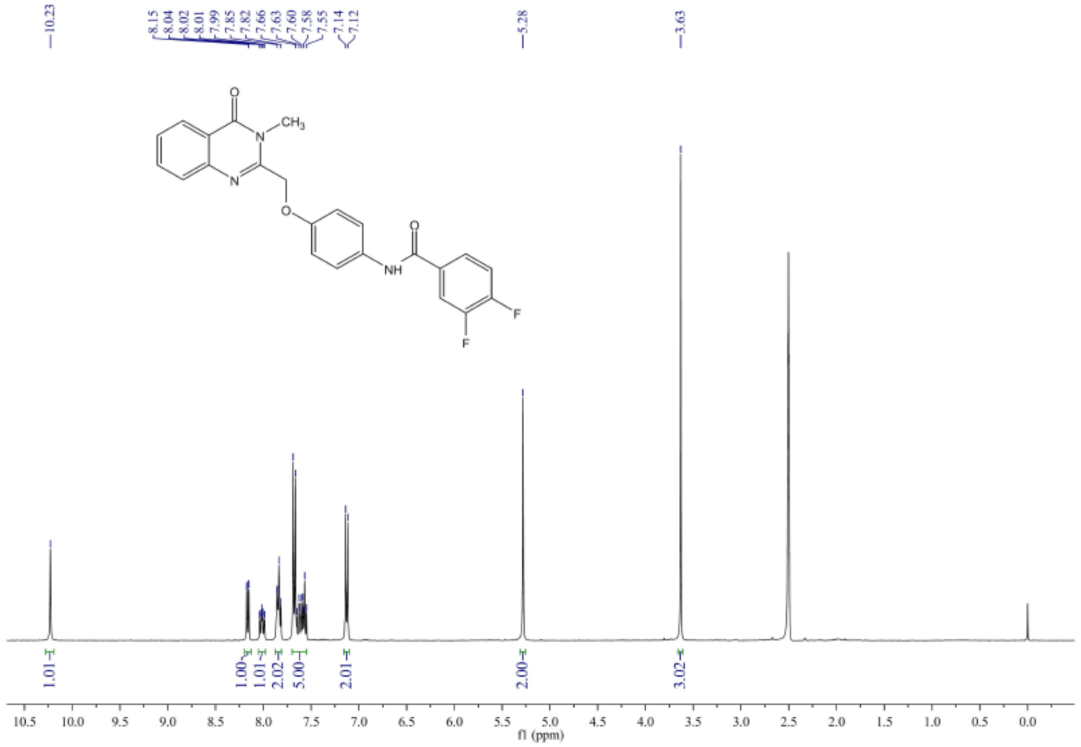

Fig.S27 ^{1}H NMR spectrogram (400 MHz, DMSO) of compound **4e**

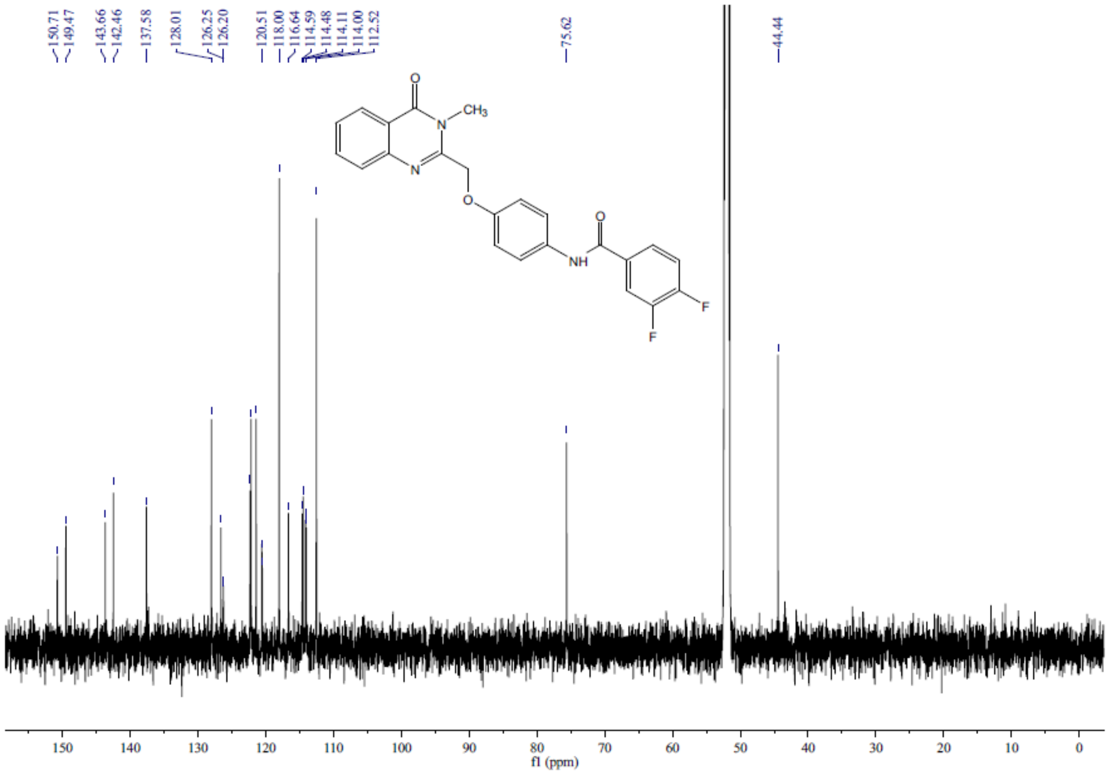

Fig.S28 ^{13}C NMR spectrogram (125 MHz, DMSO) of compound **4e**

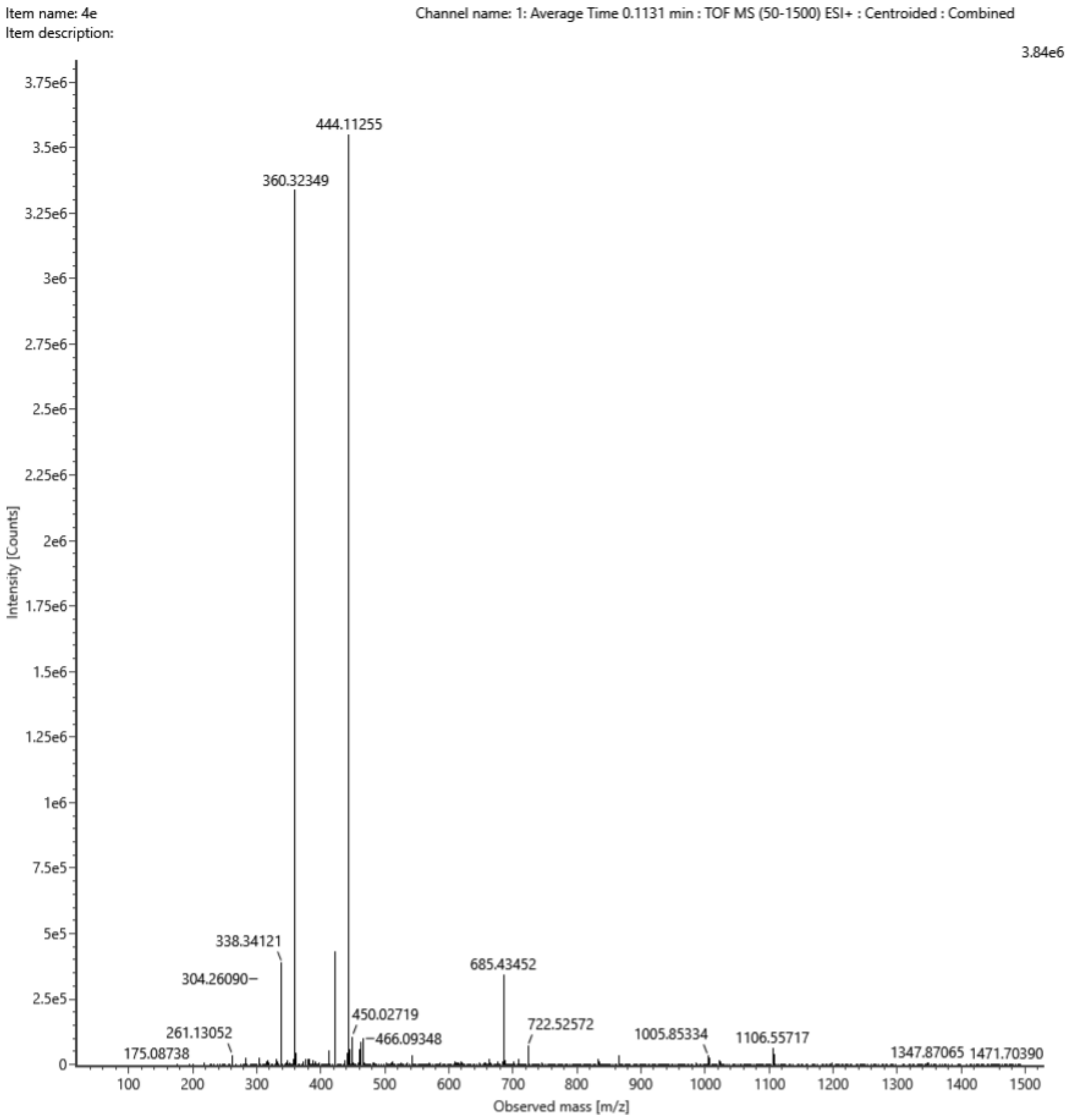

Fig.S29 HR-MS (ESI) spectrogram of compound **4e**

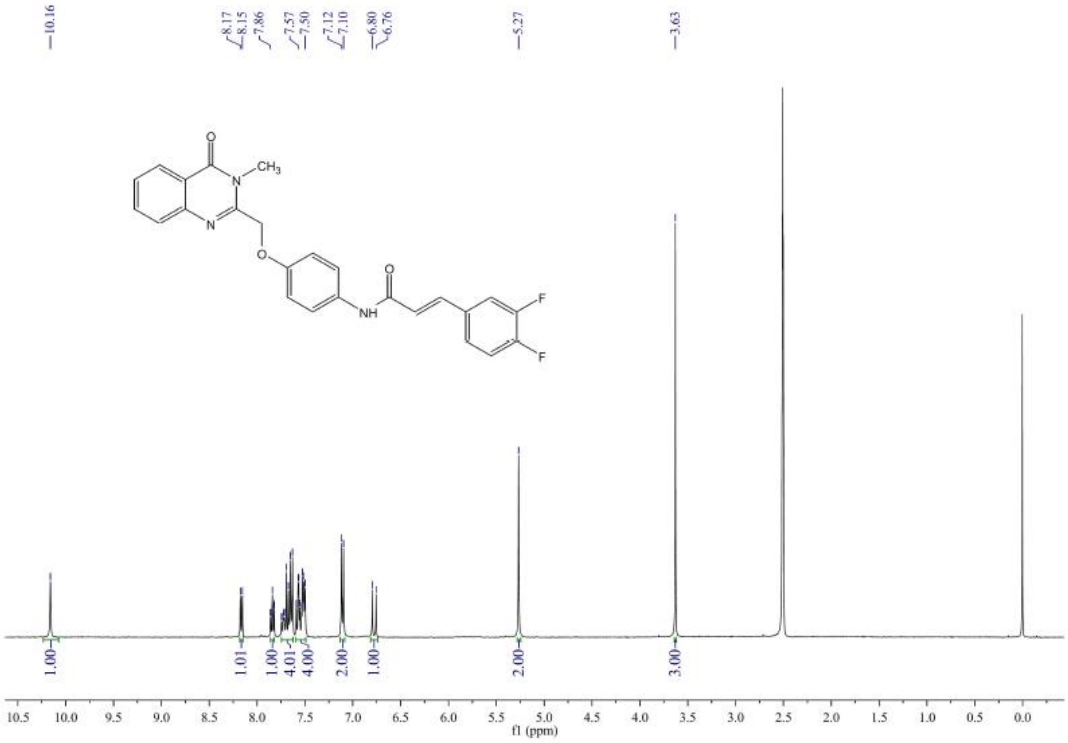

Fig.S30 ^{1}H NMR spectrogram (400 MHz, DMSO) of compound **4f**

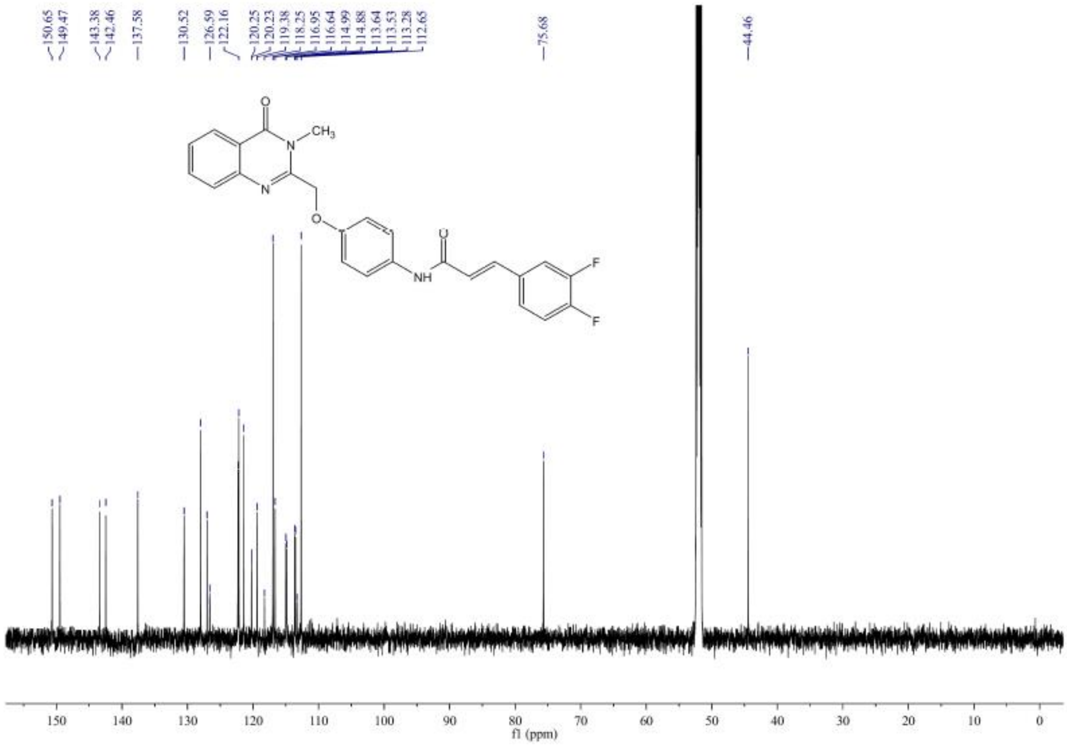

Fig.S31 ^{13}C NMR spectrogram (125 MHz, DMSO) of compound **4f**

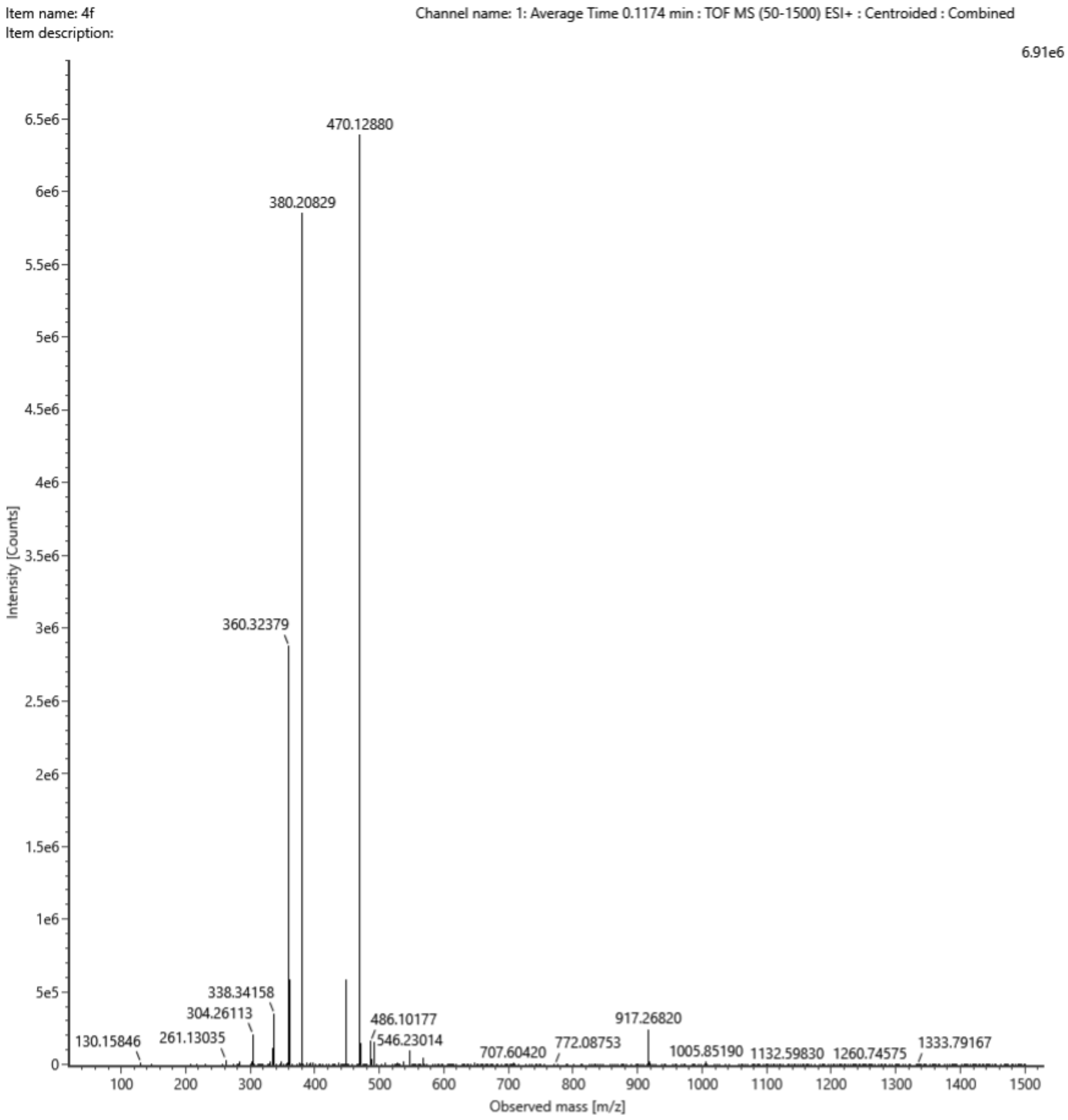

Fig.S32 HR-MS (ESI) spectrogram of compound **4f**

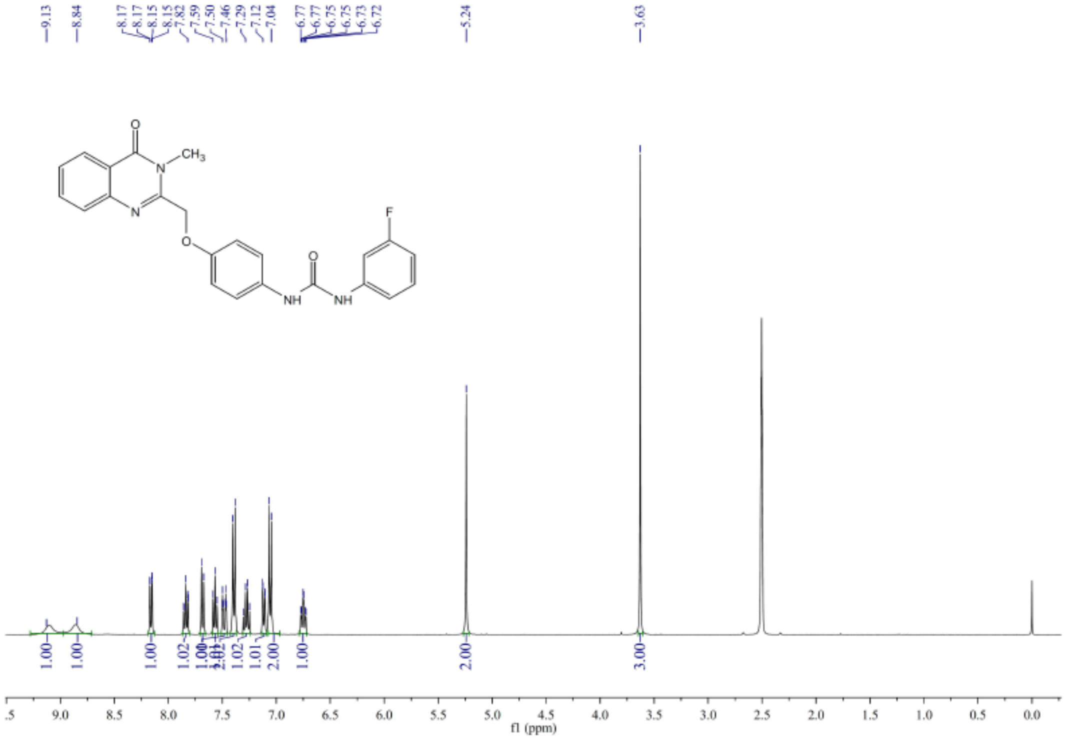

Fig.S33 ^{1}H NMR spectrogram (400 MHz, DMSO) of compound **4g**

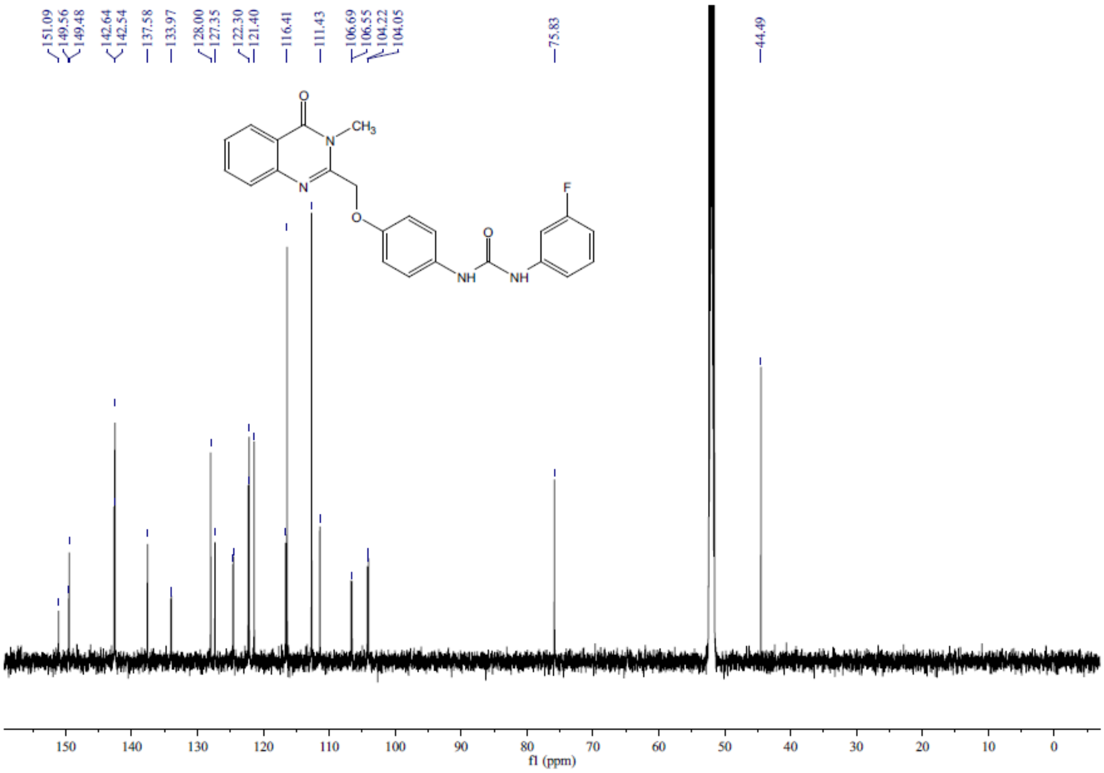

Fig.S34 ^{13}C NMR spectrogram (125 MHz, DMSO) of compound **4g**

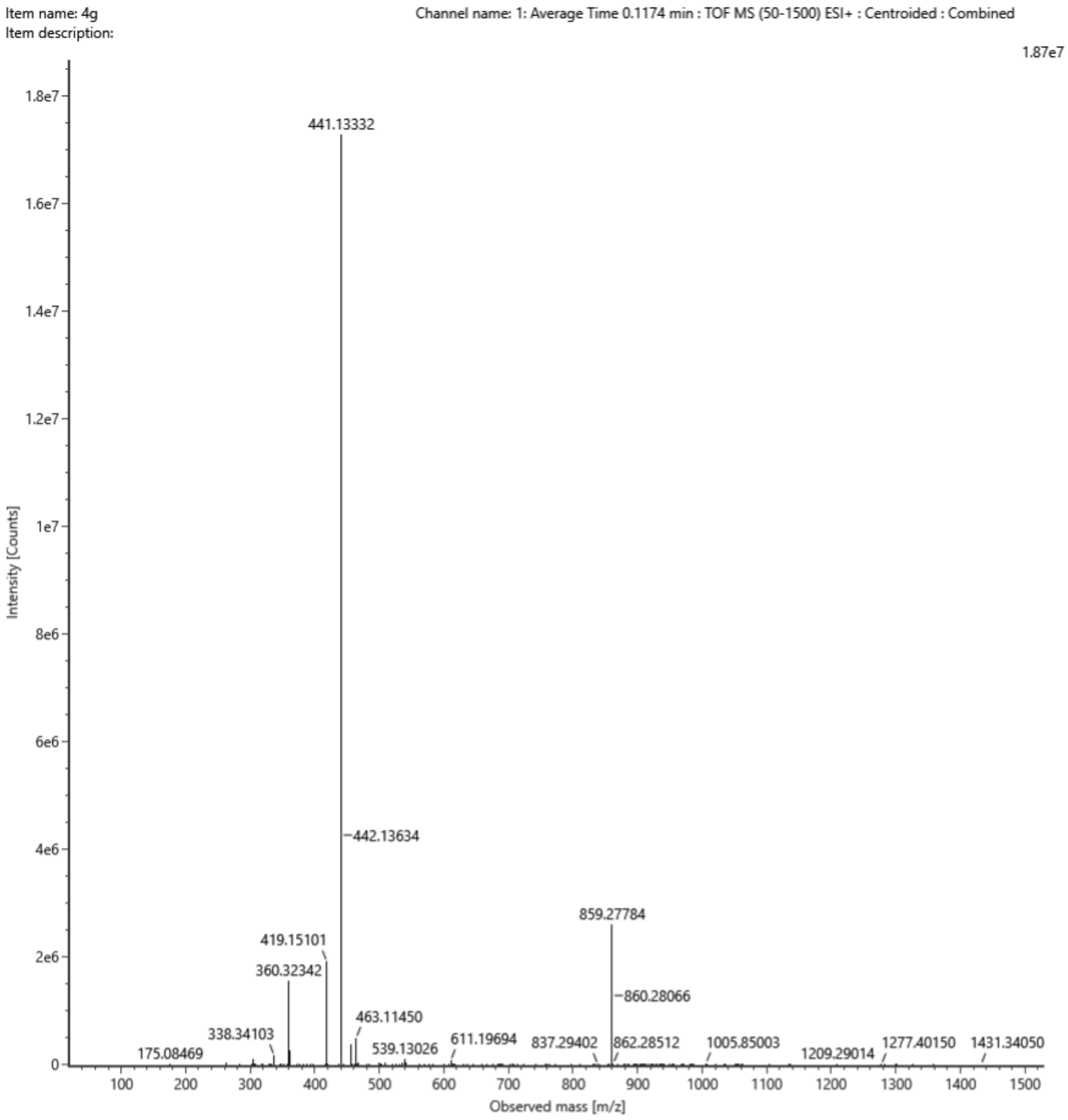

Fig.S35 HR-MS (ESI) spectrogram of compound **4g**
